# Quiescent Fibroblasts Exhibit High Metabolic Activity

**DOI:** 10.1371/journal.pbio.1000514

**Published:** 2010-10-19

**Authors:** Johanna M. S. Lemons, Xiao-Jiang Feng, Bryson D. Bennett, Aster Legesse-Miller, Elizabeth L. Johnson, Irene Raitman, Elizabeth A. Pollina, Herschel A. Rabitz, Joshua D. Rabinowitz, Hilary A. Coller

**Affiliations:** 1 Department of Chemistry, Princeton University, Princeton, New Jersey, United States of America; 2 Lewis Sigler Institute for Integrated Genomics, Princeton University, Princeton, New Jersey, United States of America; 3 Department of Molecular Biology, Princeton University, Princeton, New Jersey, United States of America; Baylor College of Medicine, United States of America

## Abstract

Many cells in mammals exist in the state of quiescence, which is characterized by reversible exit from the cell cycle. Quiescent cells are widely reported to exhibit reduced size, nucleotide synthesis, and metabolic activity. Much lower glycolytic rates have been reported in quiescent compared with proliferating lymphocytes. In contrast, we show here that primary human fibroblasts continue to exhibit high metabolic rates when induced into quiescence via contact inhibition. By monitoring isotope labeling through metabolic pathways and quantitatively identifying fluxes from the data, we show that contact-inhibited fibroblasts utilize glucose in all branches of central carbon metabolism at rates similar to those of proliferating cells, with greater overflow flux from the pentose phosphate pathway back to glycolysis. Inhibition of the pentose phosphate pathway resulted in apoptosis preferentially in quiescent fibroblasts. By feeding the cells labeled glutamine, we also detected a “backwards” flux in the tricarboxylic acid cycle from α-ketoglutarate to citrate that was enhanced in contact-inhibited fibroblasts; this flux likely contributes to shuttling of NADPH from the mitochondrion to cytosol for redox defense or fatty acid synthesis. The high metabolic activity of the fibroblasts was directed in part toward breakdown and resynthesis of protein and lipid, and in part toward excretion of extracellular matrix proteins. Thus, reduced metabolic activity is not a hallmark of the quiescent state. Quiescent fibroblasts, relieved of the biosynthetic requirements associated with generating progeny, direct their metabolic activity to preservation of self integrity and alternative functions beneficial to the organism as a whole.

## Abstract 2

Metabolomics technology reveals that fibroblast that have exited the proliferative cell cycle nevertheless utilize glucose throughout central carbon metabolism and rely on the pentose phosphate pathway for viability.

## Author Summary

Many cells in the human body are in a reversible state of quiescence, where they have exited the cell cycle but retain the capacity to re-enter it and divide again. Previous experiments in lymphocytes had suggested that quiescent cells reduce their glucose uptake and metabolic rate. In our studies, we have investigated the metabolism of fibroblasts, cells found in connective tissue and skin. Using “metabolomics” to monitor flux through metabolic pathways, we discovered that fibroblasts remain highly metabolically active even though they are not dividing. They degrade and resynthesize protein and fatty acid, and secrete large amounts of protein into the extracellular environment. Despite our expectation that quiescent cells would not have a high demand for nucleotide biosynthesis, we found that they do divert glucose to the pentose phosphate pathway, presumably to generate NADPH. The NADPH created may help the quiescent fibroblasts to detoxify free radicals or to synthesize fatty acids. Experiments in which we inhibited the pentose phosphate pathway resulted in increased apoptosis in quiescent cells, suggesting a possible strategy for selectively killing nondividing cells.

## Introduction

Proliferating and quiescent cells are expected to have vastly different metabolic needs. Proliferating cells must replicate the entirety of their cellular contents in order to divide. As a result, much of the metabolic energy in a proliferating cell is devoted to synthesizing DNA, proteins, and lipids. Quiescent cells are relieved of this massive metabolic requirement since they are not dividing and, in several well-studied model systems, they decrease their metabolic rates in accordance with their decreased proliferation rates. Yeast cultures, for instance, enter stationary phase when liquid cultures are grown to saturation in rich medium [1]. Within this population, the quiescent yeast cells fail to accumulate mass and volume [2], in part because quiescent yeast cells induce autophagy, or self-cannibalism [3]. In addition, the overall transcription rate is three to five times slower in stationary-phase than in logarithmic-phase cultures [4], and protein synthesis is reduced to approximately 0.3% of the rate in logarithmically growing cultures [5]. Therefore, the quiescent cells within a stationary-phase culture of yeast likely represent an example of a quiescent cell that has significantly reduced its metabolic activity.

Lymphocytes also undergo a major metabolic shift upon transitioning between proliferation and quiescence. Early studies showed that lectin stimulation of lymphocytes led to increased glucose uptake, and an increased rate of glycolysis and pentose phosphate pathway (PPP) activities [6],[7]. More recent experiments have focused on a murine pro-B cell lymphoid cell line, FL5.12, that proliferates in response to the cytokine interleukin IL-3 [8]. IL-3 stimulation results in an 8-fold increased glycolytic flux. IL-3 also induces the cells to consume less oxygen per glucose consumed, and to excrete much more lactate, indicating a shift away from oxidative toward glycolytic metabolism. For human peripheral blood T lymphocytes, stimulation resulted in a 30-fold increase in glycolysis [9]; for thymocytes, the increase was 50-fold [10]. These differences in quiescent and proliferating lymphocytes have played a pivotal role in our understanding of the quiescent state, and experiments with lymphocytes as a model system have been important contributors to the development of the idea that quiescence is characterized by decreased metabolic activity. Lymphocytes, however, are relatively unique among mammalian cells in having primarily a “watching and waiting” function when quiescent and performing much of their physiological role only after activation.

Our studies focus on newborn dermal fibroblasts as a model system of quiescence [11]–[13]. In vitro, primary fibroblasts isolated directly from newborn foreskin can be induced into reversible quiescence by serum withdrawal or contact inhibition. Unlike most primary cells, fibroblasts remain healthy in culture in a quiescent state for as long as 30 d with little apoptosis or senescence, and can then re-enter the cell cycle [13]. In vivo, quiescent fibroblasts are central to normal physiology as the primary synthesizers of extracellular matrix necessary for the formation of cellular tissues. In response to a wound, fibroblasts enter the cell cycle from quiescence, proliferate, and secrete a collagen-rich extracellular matrix [14], pro-angiogenesis factors that recruit new blood vessels [13], and other molecules that facilitate the wound healing response [15]. A better understanding of the transition between proliferation and quiescence in fibroblasts would have broad implications for physiology and medicine. Scarring and fibrosis result from excessive fibroblast proliferation and secretion of extracellular matrix during and after wound healing [16],[17]. Additionally, tumors may contain quiescent cells that contribute to cancer dormancy [18],[19]. Thus, a better understanding of the transition between proliferation and quiescence, including the metabolic changes that occur, could have implications for a wide range of medical conditions.

The emerging field of metabolomics promises to augment our understanding of mammalian cell physiology through the systems-level characterization of cell-wide metabolite concentrations and fluxes. Using liquid chromatography–triple quadrupole mass spectrometry, we have developed a methodology for monitoring the pool size and turnover of a large number of metabolites simultaneously [20]–[22]. Here we apply metabolomic technology, flux analysis, and biochemical assays to investigate metabolic changes after primary dermal fibroblasts enter quiescence. We discovered that contact-inhibited primary fibroblasts remain highly metabolically active while adjusting their metabolic emphasis to produce NADPH, steadily renew their proteins and lipids, and enhance secretion of specific extracellular matrix proteins.

## Results

### A Model for Cellular Quiescence in Primary Fibroblasts

**Figure 1:**
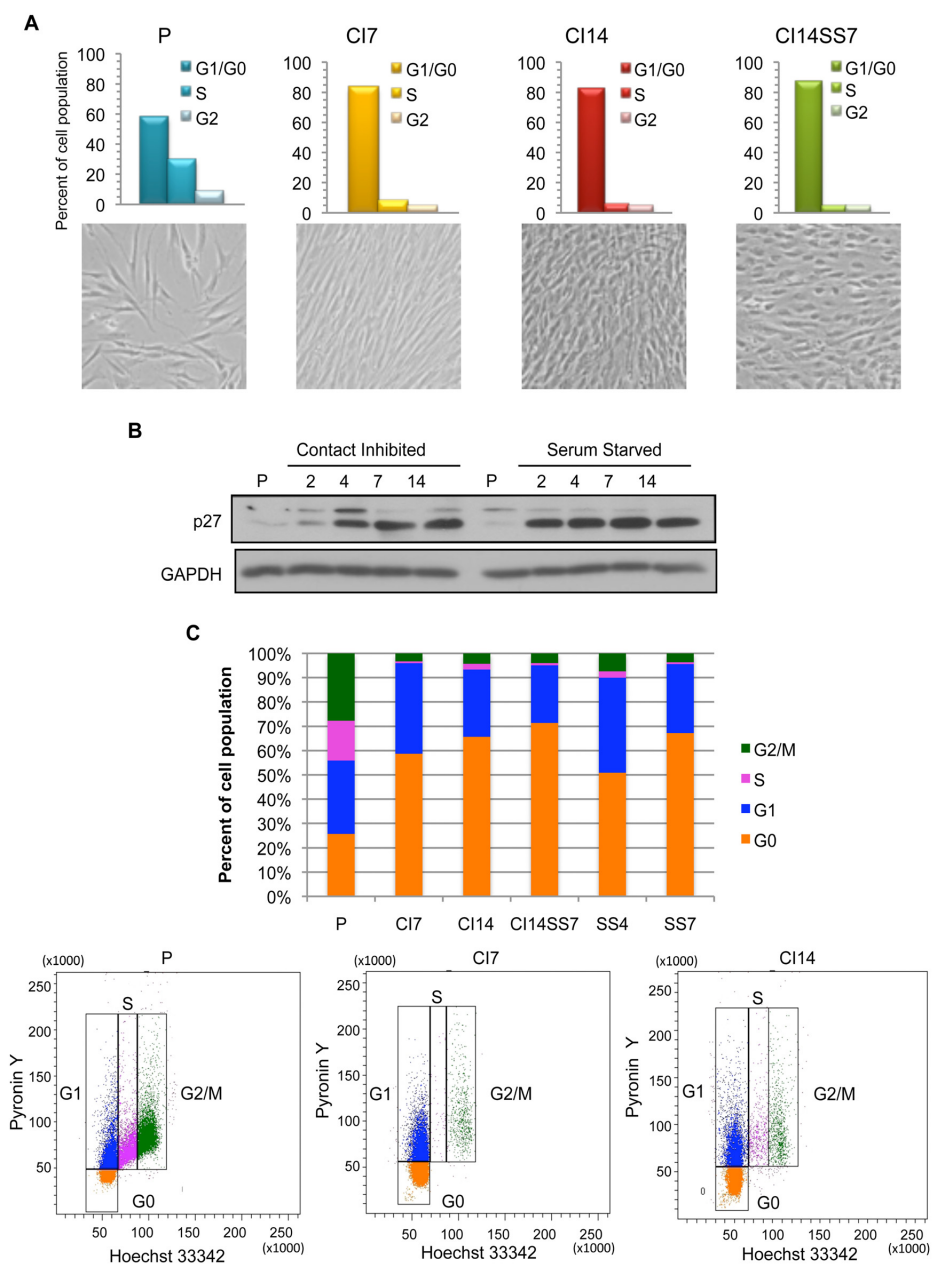
Model system of proliferating and quiescent fibroblasts. (A) Proliferating (P), CI7, CI14, and CI14SS7 fibroblasts were stained with PI and analyzed for cell cycle distribution with flow cytometry. The fraction of cells in G_0_/G_1_ increased in quiescent cells. Images of cells in different proliferative states are shown below. (B) Lysates from fibroblasts induced into quiescence by contact inhibition or serum starvation were collected over a time course and analyzed by immunoblotting with an antibody to p27^Kip1^. p27^Kip1^ levels were induced in cells made quiescent by either antiproliferative signal. (C) Proliferating and quiescent fibroblasts were stained with pyronin Y and Hoechst 33342 and analyzed by flow cytometry (lower panels). The fraction of cells with low pyronin Y content increased in fibroblasts induced into quiescence by multiple methods (upper panel).

We have developed a model system that allows us to monitor metabolic differences between proliferating and quiescent cells. Primary dermal fibroblasts were expanded and analyzed while actively proliferating, after 1 wk of growth to confluence (contact-inhibited for 7 d [CI7]), after 2 wk of confluence (contact-inhibited for 14 d [CI14]), or after 2 wk of confluence with serum concentrations decreased for the final week from 10% to 0.1% (contact-inhibited for 14 d and serum-starved for 7 d [CI14SS7]). Alternatively, fibroblasts were plated sparsely so that they did not touch each other and induced into quiescence by serum starvation and monitored after 4 d (serum-starved for 4 d [SS4]) or 7 d (serum-starved for 7 d [SS7]). In quiescent fibroblasts, the fraction of cells with 2N DNA content increased so that 80% or more of the cells were in the G_0_/G_1_ phase of the cell cycle (Figure 1A). The fraction of cells in S phase was significantly reduced, indicating that very few cells were actively dividing under these conditions. In both contact-inhibited and serum-starved fibroblasts, levels of the cyclin-dependent kinase inhibitor p27^Kip1^ were upregulated, as expected for cells that entered quiescence (Figure 1B) [23]. In addition, staining with pyronin Y for total RNA indicated that the fraction of cells with low pyronin Y, interpreted as cells in G_0_ [24], increased in fibroblasts induced into quiescence by all of these methods (Figure 1C). Pyronin Y labeling data indicate that in the contact-inhibited and serum-starved cell populations investigated as quiescence models, approximately 60%–75% of the cells are in G_0_ and most of the remainder are in G_1_.

### Rapid Glycolytic Flux in Proliferating and Quiescent Fibroblasts

**Figure 2:**
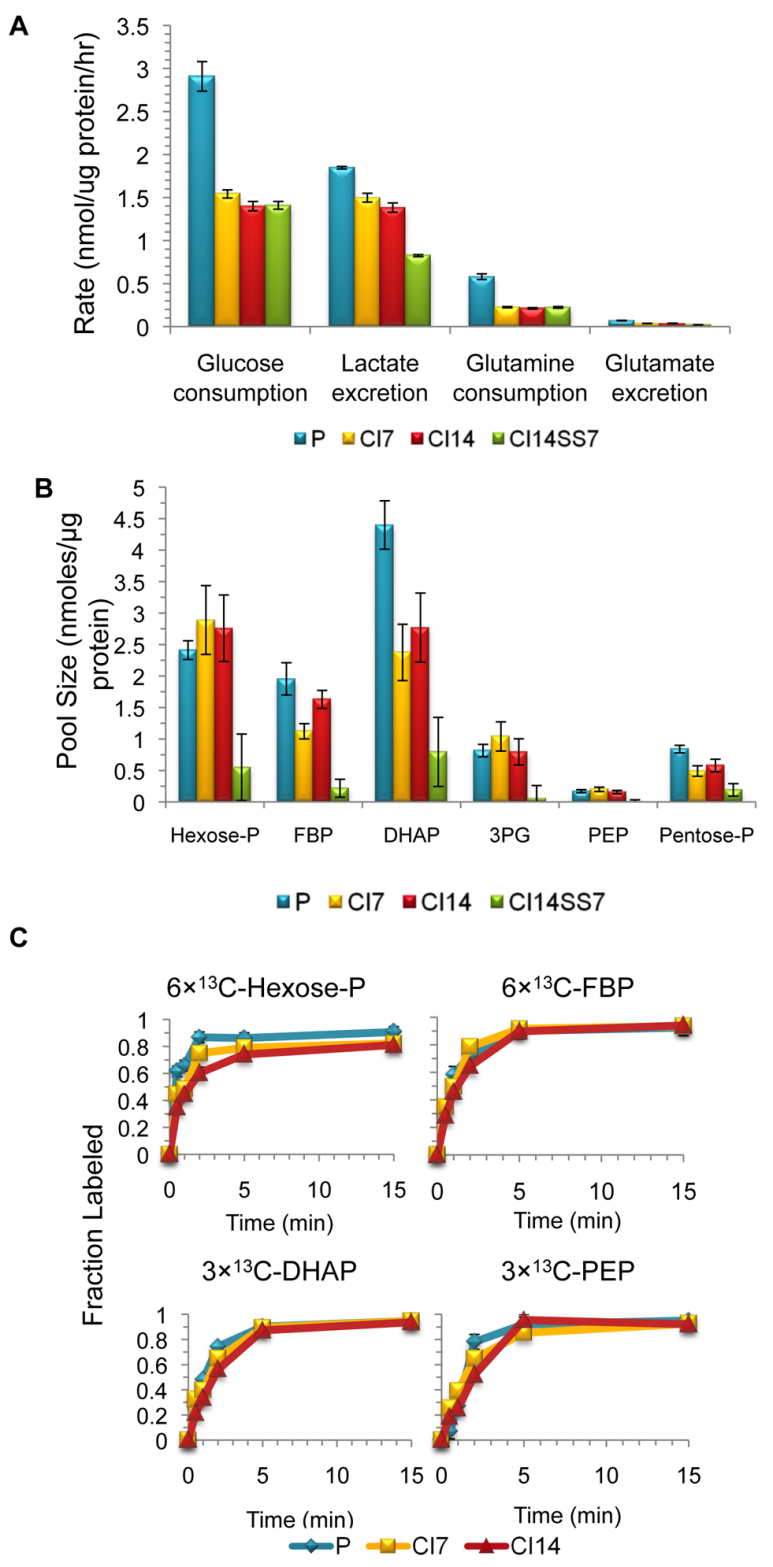
Glycolytic rates are similar in proliferating and quiescent fibroblasts. (A) The amount of glucose, lactate, glutamine, and glutamate were measured in conditioned medium from proliferating (P), CI7, CI14, and CI14SS7 cells. Data are from three experiments with five replicates at each time point, and error bars indicate standard error. (B) Metabolites were extracted from cells in different proliferative states, and the levels of specific metabolites were quantified using mass spectrometry. Metabolite levels in individual samples were normalized to protein content at the time of harvest. Means from four experiments, each containing 4–5 replicates, are shown. Error bars indicate standard error. With a false discovery rate of 0.05, none of the metabolite levels are different between the proliferating, CI7, and CI14 cells. (C) Isotope labeling dynamics of glycolysis in proliferating, CI7, and CI14 fibroblasts. Medium was changed to [U-^13^C]-glucose at time zero, and the fraction of each metabolite that is ^13^C-labeled was determined at the indicated times after switching to labeled medium. Data are pooled from five experiments, and error bars indicate standard deviations. 3PG, 3-phosphoglycerate; Hexose-P, hexose phosphate; Pentose-P, pentose phosphate; PEP, phosphoenolpyruvate.

Previous studies have reported that lymphocytes induced to exit the cell cycle in response to mitogen withdrawal exhibit decreased glycolytic activity [8]. We used several methods to assess metabolic rates in proliferating, CI7, CI14, and CI14SS7 cells. We monitored the rates at which glucose and glutamine were consumed from the medium, and lactate and glutamate were secreted into the medium. As shown in Figure 2A, the rate of glucose consumption was approximately 2-fold lower in the contact-inhibited than in the proliferating fibroblasts. Lactate secretion decreased less than 2-fold with contact inhibition alone, and roughly 2-fold with additional serum deprivation. Glucose consumption actually slightly increased in fibroblasts induced into quiescence by serum starvation (without contact inhibition) for 4 or 7 d (Figure S1). We also monitored metabolic rates in fibroblasts cultured in medium conditions containing physiological levels of glucose and glutamine (1 g/l glucose and 0.7 mM glutamine compared with 4.5 g/l glucose and 4 mM glutamine in Dulbecco's Modified Eagle Medium [DMEM]) [25],[26]. Metabolic rates were somewhat lower in proliferating fibroblasts in these low glucose/low glutamine conditions compared with proliferating fibroblasts in standard medium (Figure S1). Quiescent fibroblasts cultured in these conditions exhibited consumption and excretion rates approximately half that of proliferating fibroblasts. Our finding that glycolytic rates are similar within a factor of two in proliferating and quiescent fibroblasts is surprising given that changes in glycolytic rate have been shown to mirror changes in proliferative rate in multiple model systems [8]–[10]. Indeed, while there is a dramatic decrease in the fraction of cells in the proliferative cell cycle, even the CI14SS7 condition resulted in only a 2-fold change in glucose consumption, much less than reported in other systems. Thus, decreased metabolic activity is not a universal hallmark of quiescence.

To further assess glycolytic rates in proliferating and contact-inhibited fibroblasts, we monitored the steady state pool sizes of glycolytic intermediates using liquid chromatography coupled to tandem mass spectrometry [20]–[22]. In total, we monitored the levels of 172 metabolites, 62 of which gave signals above background in proliferating, CI7, and CI14 fibroblasts. Metabolite levels were normalized per microgram of protein in cells plated at the same density because quiescent fibroblasts are smaller and contain less protein per cell than proliferating fibroblasts (E. M. Haley, A. L.-M., and H. A. C., unpublished data). The ratio of metabolite levels in the contact-inhibited (CI7 and CI14) to proliferating fibroblasts was determined for each metabolite. Some metabolites were present at consistently higher levels in proliferating fibroblasts, while others were enriched in contact-inhibited fibroblasts, although the magnitude of these changes in metabolite levels was generally modest (Figure S2).

Levels of five glycolytic intermediates and pentose-5-phosphate (a combination of ribose-5-phosphate, ribulose-5-phosphate, and xylulose-5-phosphate, which could not be reliably differentiated in our liquid chromatography–tandem mass spectrometry [LC-MS/MS] method) are shown in Figure 2B. No statistically significant differences were observed in the levels of glycolytic intermediates between contact-inhibited (CI7 or CI14) and proliferating fibroblasts at a false discovery rate of 0.05. Some glycolytic metabolites were present at lower levels in contact-inhibited, serum-deprived (CI14SS7) fibroblasts. Thus, the transition between proliferation and quiescence induced by contact inhibition alone has little effect on the pool sizes of glycolytic metabolites in primary fibroblasts. While pool sizes are not a direct indication of changes in flux, the constant levels of glycolytic metabolites in proliferating, CI7, and CI14 fibroblasts are consistent with our finding that there is little change in the rate of glucose uptake or lactate secretion among fibroblasts in these different states.

To more directly assess the rate of flux through glycolytic pathways, we incubated fibroblasts with [U-^13^C]-glucose and determined how quickly the label was incorporated into glycolytic intermediates (Figure 2C). For hexose phosphate (a combination of glucose-1-phosphate, glucose-6-phosphate, and fructose-6-phosphate), fructose-1,6-bisphosphate (FBP), dihydroxyacetone phosphate (DHAP), and phosphoenolpyruvate, the unlabeled pools of intermediates were converted into fully ^13^C-labeled intermediates at a similar rate in proliferating, CI7, and CI14 fibroblasts.

**Figure 3:**
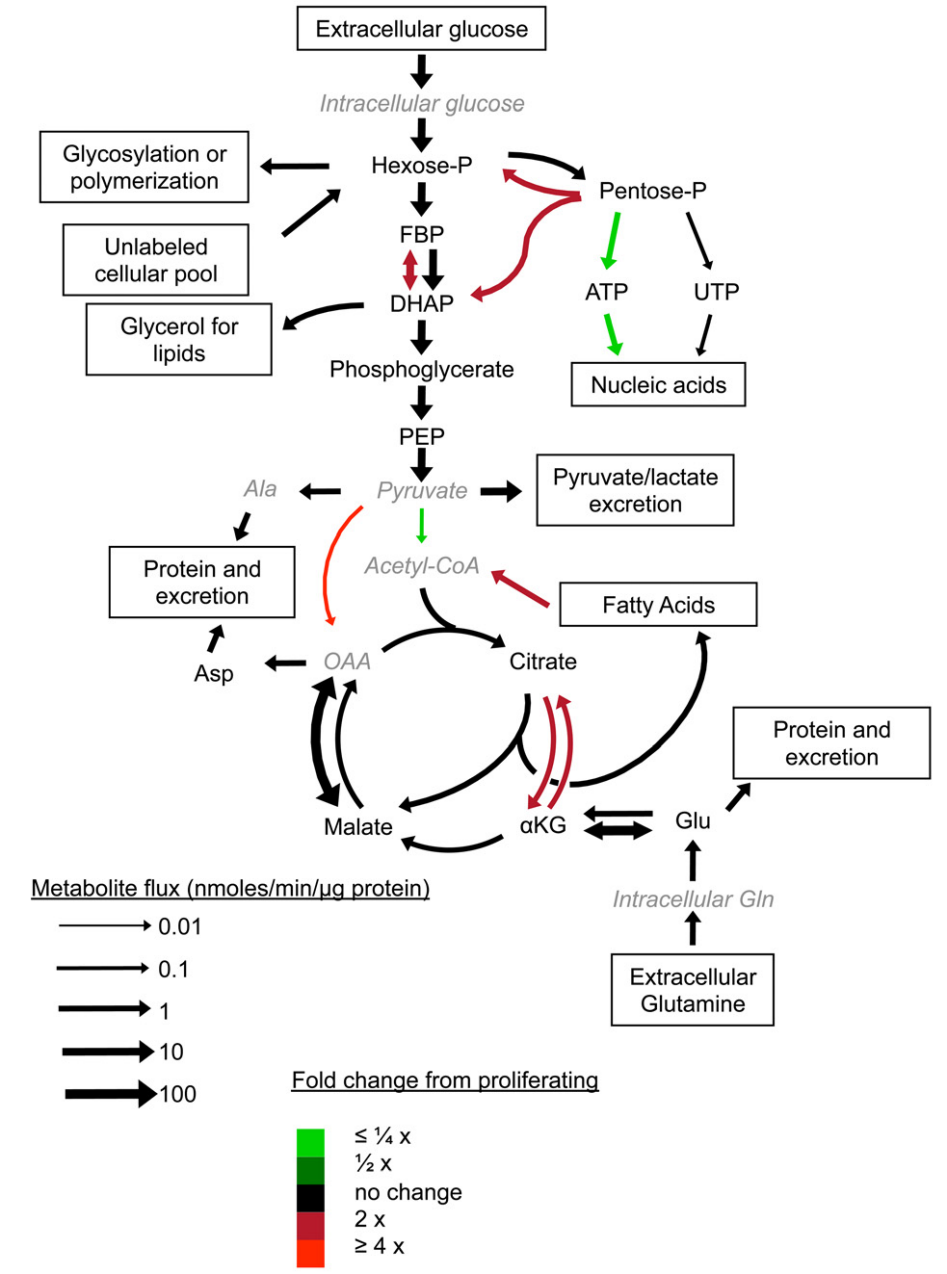
Comparison of central metabolic fluxes in proliferating and CI14 fibroblasts. Fluxes were derived by computational integration of all available experimental data within a systems-level, flux-balanced metabolic model. Arrow size indicates the magnitude of the flux in CI14 fibroblasts. Color indicates relative rates compared to proliferating fibroblasts; red indicates higher flux in CI14 fibroblasts and green indicates higher flux in proliferating fibroblasts. While the ribose phosphate–to-UTP flux is mostly faster (within the 1,000 identified solutions) for proliferating than quiescent fibroblasts, its distributions do overlap across different proliferative states, so our stringent condition for different rates is not met in this particular case (see Materials and Methods). αKG, α-ketoglutarate; Hexose-P, hexose phosphate; OAA, oxaloacetate; Pentose-P, pentose phosphate; PEP, phosphoenolpyruvate.

We also developed a computational model based on ordinary differential equations (ODEs) of central carbon metabolism for the proliferating, CI7, CI14, and CI14SS7 fibroblasts. The ODEs in the model quantify the isotope labeling dynamics of the relevant metabolites after switching into ^13^C-labeled carbon sources (Figure S3). Model parameters (i.e., metabolic fluxes and some unmeasured pool sizes) were identified by fitting all of the available laboratory data (labeling dynamics, pseudo-steady-state labeling patterns, measured pool sizes, and uptake and excretion rates). This systems-level approach enabled quantitation of flux through different metabolic pathways in proliferating, CI7, CI14, and CI14SS7 fibroblasts (Figure S4 and Table S1). For glycolysis, the inferred fluxes from hexose phosphate to FBP, and from DHAP to 3-phosphoglycerate, were similar in proliferating, CI7, and CI14 conditions (Figures 3 and S4 and Table S1; see Materials and Methods for information regarding statistical significance). In CI14SS7 fibroblasts, hexose phosphate–to-FBP and DHAP-to–3-phosphoglycerate fluxes are approximately half those in the other conditions (Figure S4 and Table S1), consistent with an approximately 2-fold reduction in glucose consumption. We conclude that glucose consumption and lactate excretion proceed rapidly in fibroblasts induced into quiescence by contact inhibition.

### Quiescent Fibroblasts Exhibit High PPP Activity

**Figure 4:**
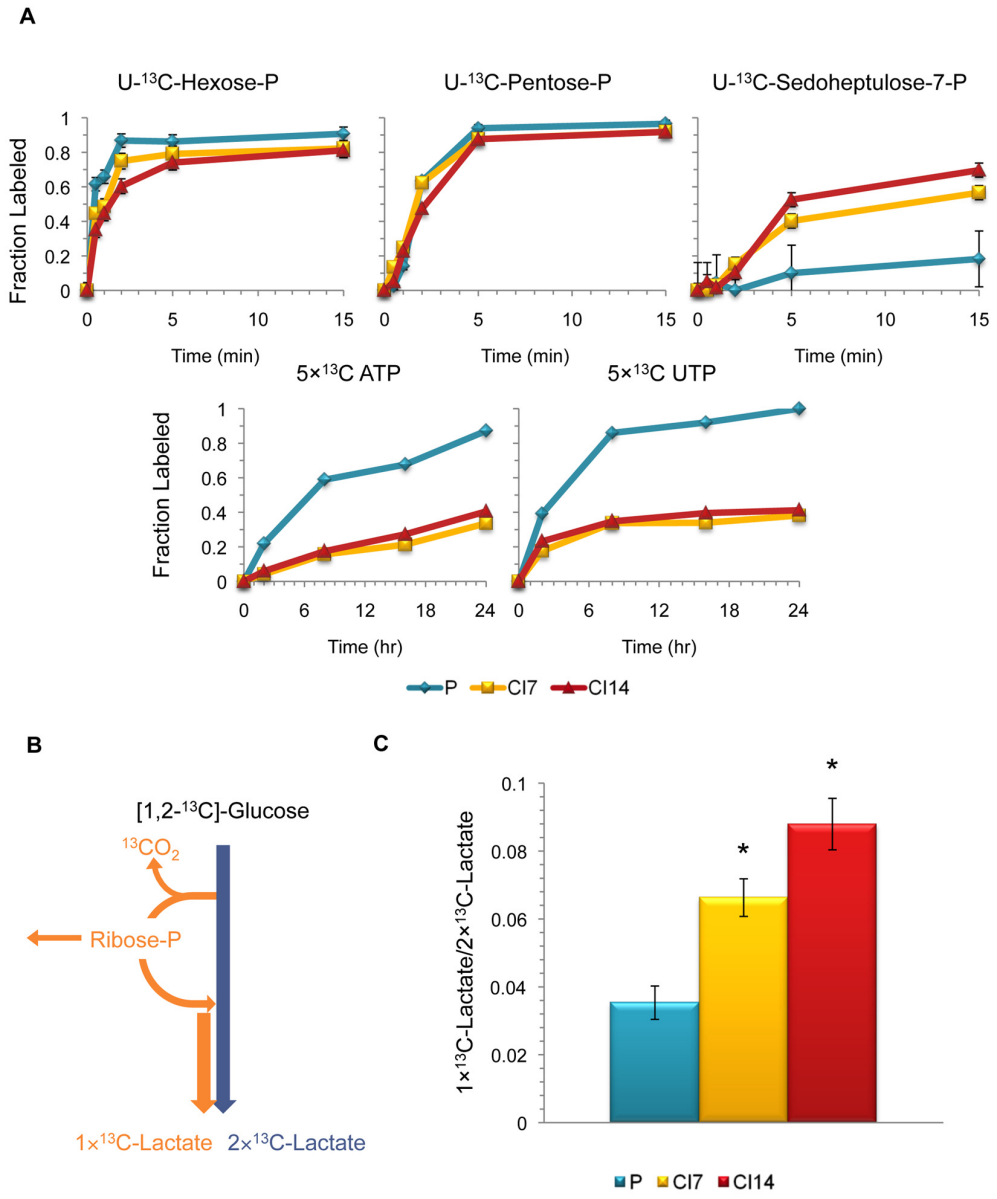
The PPP is active in quiescent fibroblasts. (A) Isotope labeling dynamics in the PPP for proliferating (P), CI7, and CI14 fibroblasts. The fraction of fully labeled hexose phosphate, pentose phosphate, or sedoheptulose-7-phosphate after addition of [U-^13^C]-glucose is plotted for cells in each condition at each time point. Similarly, the fraction of ATP and UTP with five ^13^C atoms is plotted. The 5×^13^C-ATP and 5×^13^C-UTP are uniformly labeled in their ribose portion and unlabeled in the base portion, as confirmed by tandem mass spectrometry analysis. Data are pooled from five experiments, and error bars indicate standard deviation. -P, phosphate. (B) Schematic diagram of lactate labeling from [1, 2-^13^C]-glucose. [1, 2-^13^C]-glucose is converted into 2×^13^C-lactate through the canonical glycolysis pathway and 1×^13^C-lactate through the PPP. (C) Fibroblasts in different proliferative conditions were incubated with [1, 2-^13^C]-glucose for 4 h. Levels of 1×^13^C-lactate and 2×^13^C-lactate were monitored with mass spectrometry. The ratio of 1×^13^C-lactate to 2×^13^C-lactate is plotted for fibroblasts in each condition. Means ± one standard error (*n* = 4) are shown. Asterisks indicate *p*-value<0.01 (proliferating versus CI7, *p* = 0.006, and proliferating versus CI14 fibroblasts *p* = 0.002 by Student's *t* test).

The PPP produces ribose-5-phosphate, needed for the biosynthesis of nucleotides, and NADPH, which can be used as a cofactor for the biosynthesis of macromolecules including fatty acids. We anticipated that proliferating cells would have higher demands for both ribose-5-phosphate and NADPH than quiescent cells, and thus higher PPP flux. Surprisingly, the pentose phosphate pool incorporated ^13^C label very rapidly in proliferating, CI7, and CI14 fibroblasts when the cells were incubated with labeled [U-^13^C]-glucose (Figure 4A). Indeed, according to our computational model, hexose phosphate–to–pentose phosphate flux was actually slightly higher in contact-inhibited (both CI7 and CI14) fibroblasts than in proliferating fibroblasts (though the effect was not statistically significant). Additional serum deprivation only slightly decreased oxidative PPP flux, with the oxidative PPP flux–to–glycolytic flux ratio highest in CI14SS7 fibroblasts. Thus, the oxidative PPP is actively utilized in both proliferating and quiescent cells.

We anticipated that ribose generated from the PPP would be incorporated into nucleotide triphosphates more rapidly in proliferating than quiescent cells because of their increased need for nucleotide triphosphates for RNA and DNA synthesis. Indeed, in proliferating fibroblasts, ATP and UTP with labeled ribose rings accumulate more rapidly in proliferating fibroblasts (Figure 4A). The results confirm that biosynthesis of nucleotides is more rapid in the proliferating cells.

Given that quiescent fibroblasts do not commit ribose phosphate to nucleotide biosynthesis, we reasoned that quiescent cells might recycle ribose phosphate back to glycolytic intermediates through the non-oxidative branch of the PPP. To test this hypothesis, we monitored the ratio of 1×^13^C-lactate to 2×^13^C-lactate after incubating the cells with [1, 2-^13^C]-glucose. As previously described [27], 1×^13^C-lactate is formed when glucose is metabolized through the oxidative portion of the PPP to ribulose-5-phosphate. In this pathway, glucose molecules lose one ^13^C atom in the form of CO_2_, and are then returned to glycolysis through the non-oxidative branch of the PPP (Figure 4B). 2×^13^C-lactate is formed by the canonical glycolysis pathway from glucose to lactate. The ratio of 1×^13^C-lactate to 2×^13^C-lactate provides an indication of the extent to which the non-oxidative branch of the PPP is utilized. This ratio is significantly higher in CI7 than proliferating fibroblasts, and even higher in CI14 fibroblasts (Figure 4C).

As another indication of the rate of flux through the non-oxidative branch of the PPP, we monitored labeling of sedoheptulose-7-phosphate, a metabolic intermediate in the non-oxidative PPP. Sedoheptulose-7-phosphate is labeled rapidly in CI7 and CI14 but not proliferating fibroblasts fed [U-^13^C]-glucose (Figure 4A), indicating higher flux through the non-oxidative branch of the PPP in quiescent cells. Our systems-level flux analysis confirmed increased flux from ribose phosphate back to glycolysis in contact-inhibited compared with proliferating fibroblasts (Figures 3 and S4 and Table S1). Thus, ribose phosphate generated from the PPP is utilized for nucleotide biosynthesis in proliferating fibroblasts but is recycled back to glycolytic intermediates in quiescent fibroblasts.

### Functional Importance of the PPP

**Figure 5:**
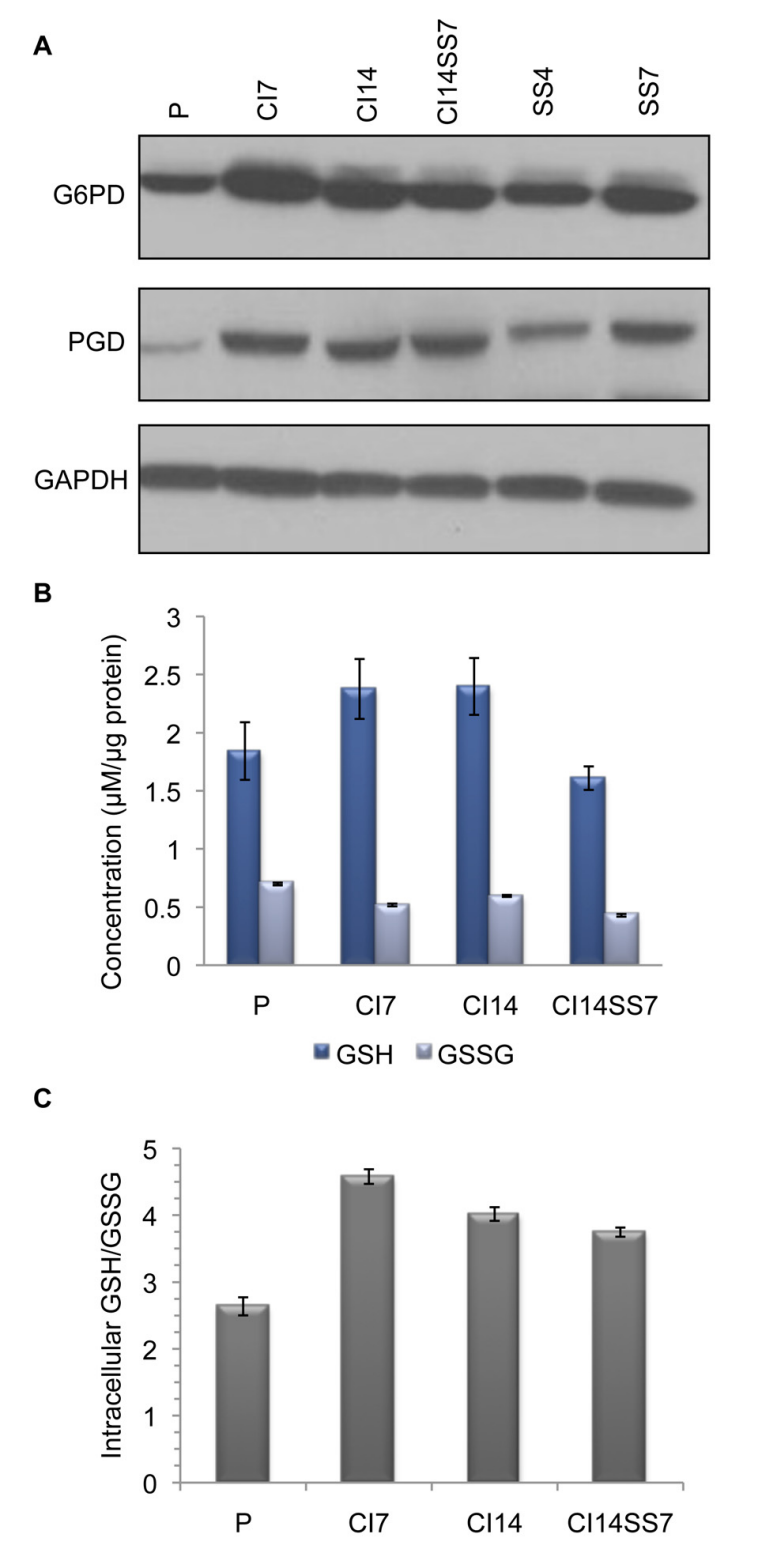
PPP enzymes are induced and the fraction of GSH is enhanced in quiescent fibroblasts. (A) Protein levels of G6PD and PGD, both of which generate NADPH, were monitored in proliferating (P) and quiescent fibroblasts by immunoblotting. GAPDH was monitored as a loading control. (B) Total GSH and GSSG content of proliferating, CI7, CI14, and CI14SS7. Data represent one experiment performed in duplicate, and error bars indicate standard deviation. (C) The ratio of GSH to GSSG was calculated using the data from (B).

To investigate the mechanistic basis for the high PPP flux in quiescence fibroblasts, we monitored protein levels of two key enzymes in the PPP, both of which generate NADPH: glucose-6-phosphate dehydrogenase (G6PD) and 6-phosphogluconate dehydrogenase (PGD). Protein levels of both G6PD and PGD were elevated in fibroblasts induced into quiescence by either contact inhibition or serum starvation in comparison to proliferating fibroblasts (Figure 5A). These results suggest that contact-inhibited and serum-starved fibroblasts may activate a program that results in increased levels of PPP enzymes.

Both proliferating and quiescent fibroblasts generate NADPH through the PPP. The NADPH may be used for biosynthesis or to regenerate the reduced forms of glutathione or thioredoxin. We monitored reduced and oxidized glutathione (GSH and GSSG, respectively) in proliferating, CI7, CI14, and CI14SS7 fibroblasts. As shown in Figure 5B and 5C, GSH was slightly increased, and the ratio of GSH to GSSG significantly enhanced, in quiescent (CI7, CI14, and CI14SS7) compared with proliferating fibroblasts. The results are consistent with a model in which quiescent fibroblasts upregulate NADPH production in part to ensure adequate GSH as protection against free radicals [28].

**Figure 6:**
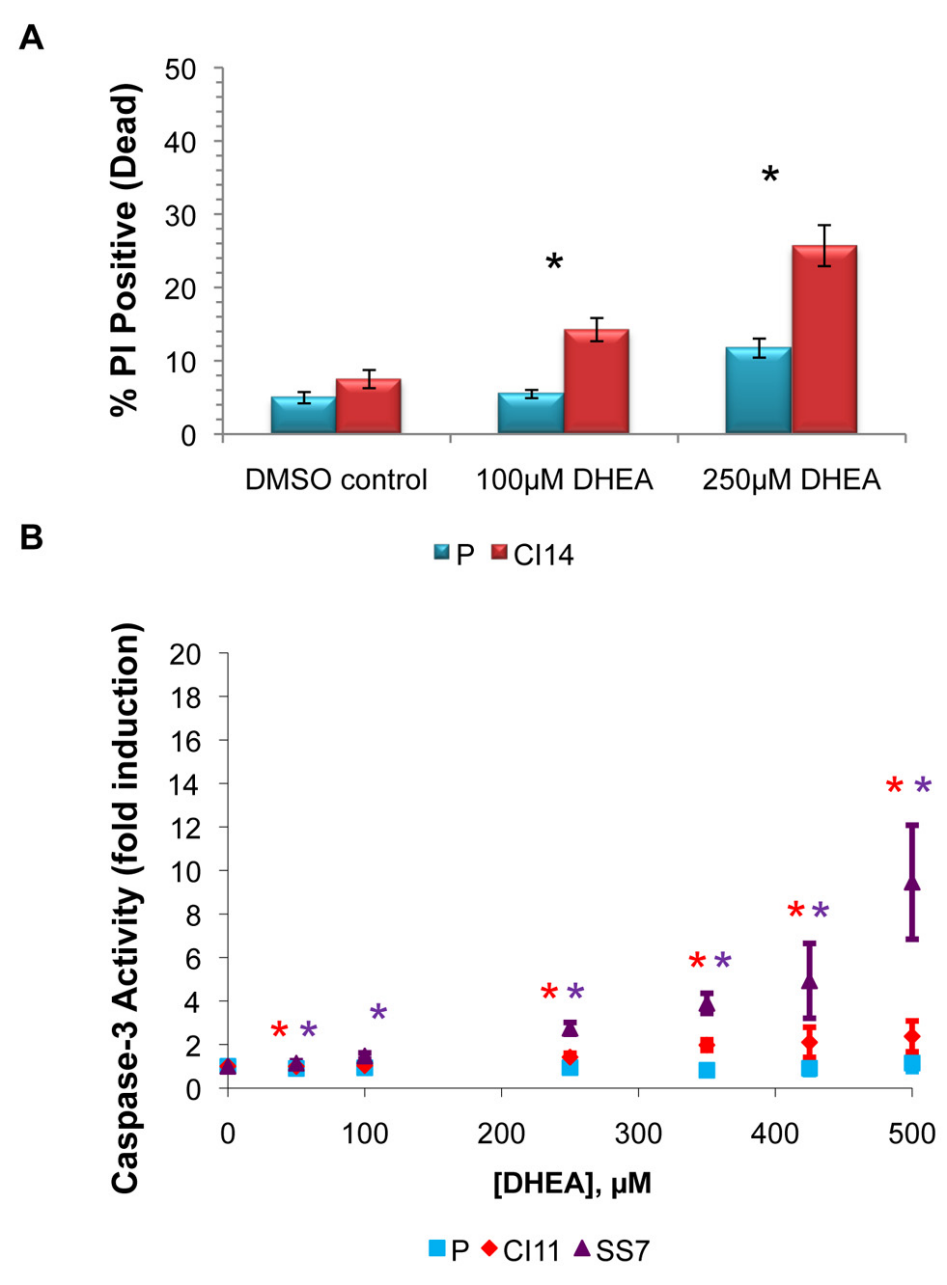
The PPP contributes to the survival of quiescent fibroblasts. (A) Proliferating (P) or CI14 fibroblasts were treated with DMSO control, 100 µM DHEA, or 250 µM DHEA for 4 d. Cells were incubated with PI and analyzed by flow cytometry. Data are from four independent experiments; error bars indicate standard error. For CI14 versus proliferating with no treatment, *p* = 0.113. For CI14 versus proliferating with 100 µM DHEA, *p* = 0.0012. For CI14 versus proliferating with 250 µM DHEA, *p* = 0.0011. Asterisks indicate statistical significance of p<0.01. (B) Proliferating fibroblasts, fibroblasts contact-inhibited for 11 d (CI11), or SS7 fibroblasts were treated with ethanol vehicle control or varying amounts of DHEA dissolved in ethanol for 4 d. Cells were analyzed for caspase-3/7 activity by monitoring luminescence emission of a caspase-3/7 substrate. Data were normalized to the vehicle control. For proliferating versus CI11 cells, results are an average of four experiments with 2–3 replicates; error bars represent standard error and asterisks indicate statistical significance of *p*<0.05. Normalized caspase-3/7 activity in CI11 and proliferating cells was statistically significantly different at all doses except 100 µM. For proliferating versus SS7 cells, data represent three experiments with three replicates in each. Normalized caspase activity in SS7 and proliferating cells was statistically significantly different at all doses.

We then tested the functional importance of the PPP in quiescent and proliferating fibroblasts. We incubated proliferating or CI14 fibroblasts with dehydroepiandrosterone (DHEA), a small molecule inhibitor of the PPP [29],[30] for 4 d and monitored the fraction of cells that were dead with propidium iodide (PI) labeling followed by flow cytometry. We discovered that the contact-inhibited fibroblasts exhibited a statistically significant increase in cell death compared with the proliferating fibroblasts from DHEA treatment at 100 µM and 250 µM doses (*p*<0.01) (Figure 6A). This result is particularly impressive given that almost all known metabolic inhibitors and cytotoxins preferentially kill proliferating cells [18],[31],[32]. Assaying for caspase-3/7 activity revealed that the mechanism of DHEA-induced cell death in the quiescent fibroblasts is via apoptosis (Figure 6B). The apoptosis-inducing effect of DHEA was significantly stronger in fibroblasts that were confluent for 11 d than in proliferating fibroblasts, and yet stronger in fibroblasts serum-starved for 7 d in the absence of contact inhibition.

### Truncated Tricarboxylic Acid Cycle in Proliferating but Not Quiescent Fibroblasts

**Figure 7:**
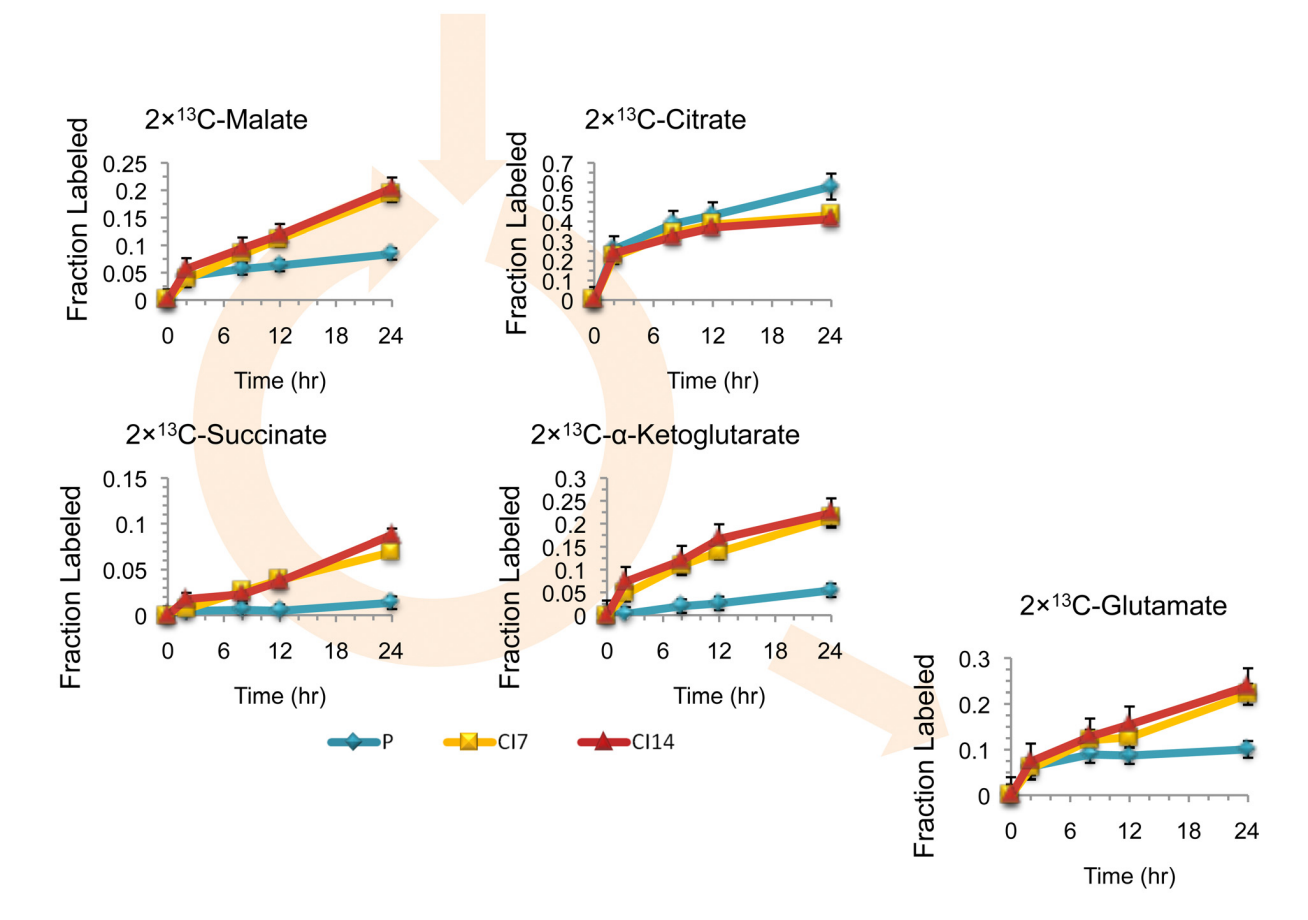
A truncated TCA cycle in proliferating but not contact-inhibited fibroblasts. Proliferating (P), CI7, and CI14 fibroblasts were switched from unlabeled to [U-^13^C]-glucose at time zero. The graphs show the fractional incorporation of ^13^C into the indicated metabolites over time. Data represent averages from three experiments, and error bars indicate standard deviation. Note the minimal labeling of α-ketoglutarate and succinate in the proliferating cells.

**Figure 8:**
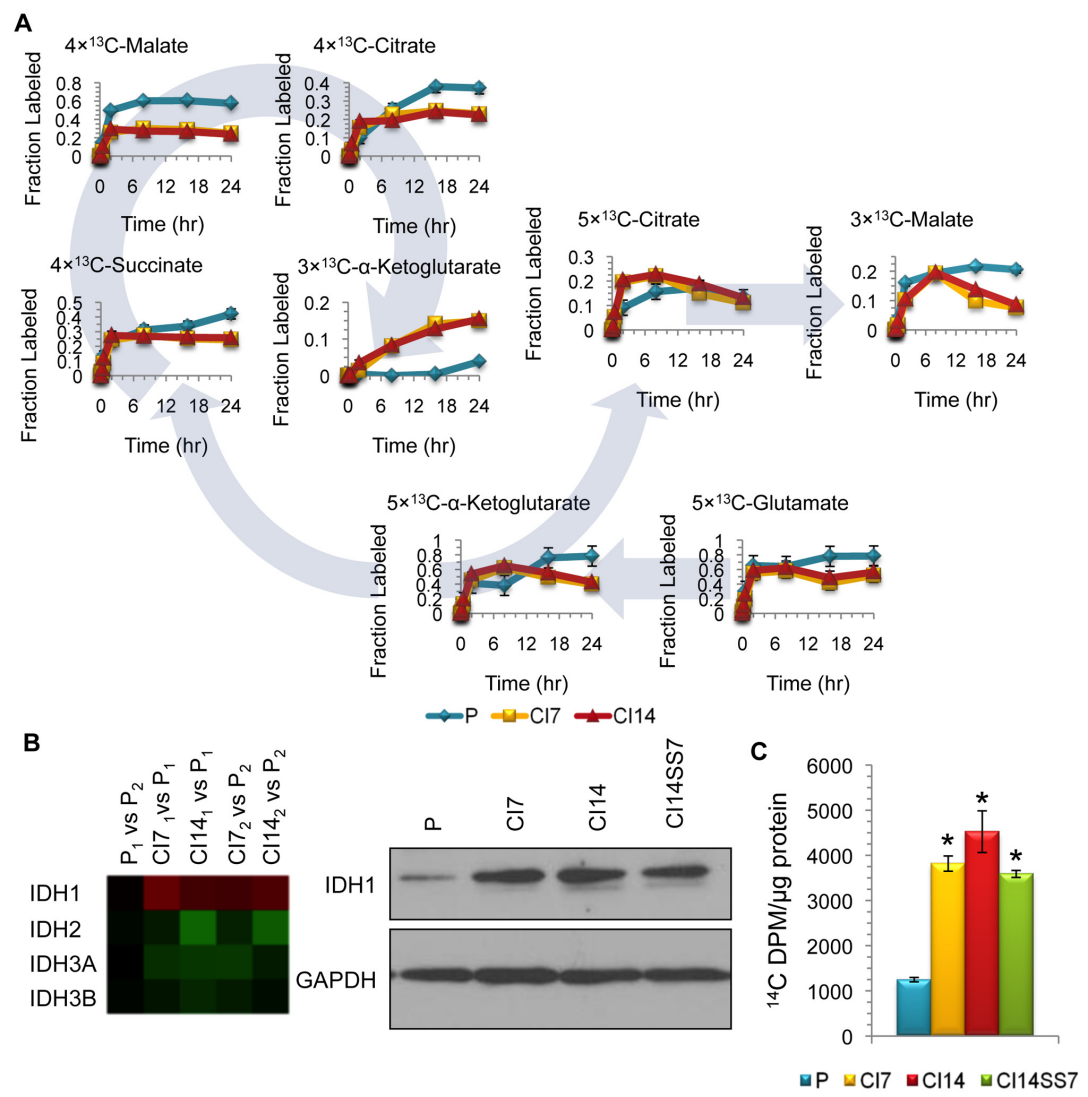
Glutamine drives both clockwise and counterclockwise flux through the TCA cycle. (A) Proliferating (P), CI7, or CI14 fibroblasts were incubated with [U-^13^C]-glutamine. Metabolites were harvested and their extent of labeling measured by LC-MS/MS. α-Ketoglutarate in the TCA cycle can be converted to succinate in the clockwise (or “forward”) direction or converted to citrate in the counterclockwise (or “reverse”) direction. The only known route to 5×^13^C-citrate is via this “reverse” flux from α-ketoglutarate. 5×^13^C-Citrate can then be converted to 3×^13^C-malate by ATP-citrate lyase to produce acetyl-CoA to drive fatty acid biosynthesis. Data represent the average of four experiments. Error bars indicate standard deviations. (B) IDH1 is upregulated at the transcript and protein level in quiescent fibroblasts. Transcript levels of IDH1 were monitored in two independent experiments (indicated with subscripts) of proliferating, CI7, and CI14 fibroblasts by microarray (left panel). Data are shown in a heatmap format with elevated expression in quiescent cells shown in red and decreased expression in quiescent cells in green. Results are shown for multiple isocitrate dehydrogenase isozymes. Protein levels for IDH1, a metabolic enzyme that catalyzes the conversion of isocitrate to α-ketoglutarate and thereby generates NADPH, were monitored by immunoblotting (right panel). GAPDH was monitored as a loading control. (C) Proliferating, CI7, CI14, and CI14SS7 fibroblasts were incubated with [U-^14^C]-glutamine for 24 h. Fatty acids were extracted, and ^14^C incorporation was determined by scintillation counting and normalized for the amount of protein present. Error bars indicate standard error. *p*-Values were determined with the Student's *t* test, and asterisks indicate *p*<0.05. For CI7 versus proliferating, *p* = 0.0025; for CI14 versus proliferating, *p* = 0.018; for CI14SS7 versus proliferating, *p* = 0.0001.

Previous studies concluded that proliferating lymphocytes actively utilize glycolytic pathways to generate ATP while quiescent lymphocytes generate energy via an influx of fatty acids and proteins that are metabolized through the tricarboxylic acid (TCA) cycle [8]. To investigate TCA cycle usage, we monitored metabolite labeling through the TCA cycle after addition of [U-^13^C]-glucose, [3-^13^C]-glucose or [U-^13^C]-glutamine in proliferating, CI7, and CI14 fibroblasts. As shown in Figure 7, proliferating and contact-inhibited fibroblasts incorporate two carbon units from glucose into citrate via acetyl-CoA at comparable rates. In CI7 and CI14 fibroblasts, the labeled carbons progress through the TCA cycle to form 2×^13^C-α-ketoglutarate, as expected. In proliferating fibroblasts, however, there is a substantial decrease in the transmission of labeled carbons from citrate to α-ketoglutarate, succinate, and malate. Experiments using [U-^13^C]-glutamine further support the truncation of the TCA cycle (Figure 8). While carbon from glutamine effectively transverses the left side of the TCA cycle in the standard clockwise direction to yield 4×^13^C-citrate in both proliferating and quiescent fibroblasts, subsequent formation of 3×^13^C-α-ketoglutarate by isocitrate dehydrogenase hardly occurs in proliferating fibroblasts. The decreased flux from citrate to α-ketoglutarate in proliferating fibroblasts was confirmed via our systems-level flux identification (Figures 3 and S4 and Table S1).

When carbon skeletons are removed from the TCA cycle for the synthesis of macromolecular precursors including amino acids, other long carbon skeletons are needed to replace them. This anaplerotic refilling should be especially important for proliferating fibroblasts since their TCA cycle activity is truncated at citrate. The major anaplerotic reaction from glycolysis involves the carboxylation of pyruvate to form oxaloacetate. This reaction can be monitored by feeding cells [3-^13^C]-glucose and monitoring the fraction of citrate or malate with label, since the ^13^C is retained only when the anaplerotic reaction via pyruvate carboxylase is utilized. Surprisingly, the ratios of 1×^13^C-citrate to unlabeled citrate and/or 1×^13^C-malate to unlabeled malate were significantly increased in CI7, CI14, and CI14SS7 fibroblasts compared with proliferating fibroblasts (Table S2). In addition, quantitative flux analysis revealed that anaplerotic flux from pyruvate to oxaloacetate is elevated in CI7, CI14, and CI14SS7 compared with proliferating fibroblasts (Figure S4 and Table S1), while the flux from pyruvate to acetyl-CoA is lower in CI14 and CI14SS7 fibroblasts than in proliferating fibroblasts. Thus, contact inhibition was associated with both an increase in canonical TCA cycle activity past citrate, and an increase in anaplerotic TCA cycle flux from pyruvate to oxaloacetate. Proliferating fibroblasts, in contrast, seem unlikely to have sufficient carbon skeletons from glucose for the production of proteogenic amino acids not present in the cell growth medium.

### Glutamine Is the Preferred Anaplerotic Source in Proliferating Fibroblasts

We hypothesized that proliferating fibroblasts rely on another source for carbon skeletons. Supplementation with glutamine has been shown to be necessary for cultured cells, especially actively proliferating cells [33]–[36]. Accordingly, we monitored the rate of glutamine consumption by proliferating, CI7, CI14, and CI14SS7 fibroblasts (Figures 2A and S1). CI7, CI14, and CI14SS7 fibroblasts consume approximately half as much glutamine per microgram of protein as proliferating fibroblasts. CI7 and CI14 fibroblasts secrete glutamate at a lower rate compared with proliferating fibroblasts, and CI14SS7 fibroblasts secrete glutamate at a lower rate than CI7 or CI14 fibroblasts. SS4 and SS7 fibroblasts, on the other hand, consume glutamine and secrete glutamate at a faster rate than proliferating fibroblasts (Figure S1). The relative rate of glutamine consumption in proliferating versus CI14 fibroblasts in low glucose/low glutamine conditions is similar to that in standard medium. As shown in Figure 8, incubation of proliferating, CI7, and CI14 fibroblasts with [U-^13^C]-glutamine results in rapid labeling of glutamate, α-ketoglutarate, succinate, malate, and citrate, indicating that glutamine is used by both proliferating and contact-inhibited fibroblasts for TCA cycle anaplerosis. Since very few glucose carbons are incorporated into the TCA cycle in proliferating fibroblasts, glutamine may serve as the major anaplerotic precursor in proliferating fibroblasts [36]–[39].

### Glutamine Labeling Reveals “Reverse” TCA Flux

[U-^13^C]-glutamine is converted into 5×^13^C-glutamate and subsequently to 5×^13^C-α-ketoglutarate. 5×^13^C-α-ketoglutarate can proceed through the TCA cycle in the forward direction to generate 4×^13^C-succinate, or, alternatively, it can be reductively carboxylated to 5×^13^C-citrate using NADPH as the electron source [40],[41]. As shown in Figure 8A, introduction of [U-^13^C]-glutamine led to conversion of approximately 15% of the citrate to the 5×^13^C form in proliferating, CI7, and CI14 fibroblasts by 8 h, with more rapid labeling in contact-inhibited fibroblasts. These results support a model in which there is both forward and reverse flux between citrate and α-ketoglutarate, with greater flux in both directions in contact-inhibited than in proliferating fibroblasts (Figures 3 and S4 and Table S1). The forward and reverse flux likely occur in different compartments, with α-ketoglutarate reductively carboxylated by isocitrate dehydrogenase 2 (IDH2) in the mitochondrion, and the resulting citrate reconverted to α-ketoglutarate by IDH1 in the cytosol [42]. As both IDH1 and IDH2 use NADP(H) as their redox cofactor, the net effect is transfer of high energy electrons in the form of NADPH to the cytosol. Consistent with greater flux through this pathway in contact-inhibited fibroblasts, IDH1 protein is increased by contact inhibition at the transcript and protein levels (Figure 8C). Thus, two major pathways to cytosolic NADPH, the PPP and the IDH2/IDH1 shuttle, are upregulated at both the protein and flux level in contact-inhibited fibroblasts.

### Fatty Acid and Protein Degradation and Resynthesis Occur Rapidly in Proliferating and Quiescent Fibroblasts

Quiescent cells do not dilute out older macromolecules, organelles, or membranes with cell division, and thus may be more dependent than proliferating cells on mechanisms to break down and resynthesize membrane components and macromolecules. Our data are consistent with increased fatty acid degradation in contact-inhibited fibroblasts. Carnitine, a metabolite involved in the transport of fatty acids from the cytoplasm to the mitochondria during fatty acid degradation, is present at higher levels in CI7 and CI14 fibroblasts than in proliferating fibroblasts (Figure S2). Also, quantitative flux identification revealed, based on long-term labeling patterns of citrate, increased fatty acid breakdown in CI7 and CI14 fibroblasts, but lower rates of fatty acid breakdown in CI14SS7 fibroblasts (Figures 3 and S4 and Table S1).

The enhanced rate of fatty acid degradation in contact-inhibited fibroblasts seems to be enabling fatty acid biosynthesis to occur at a similar rate in proliferating and contact-inhibited fibroblasts. During fatty acid synthesis, citrate is transported out of the mitochondria to the cytoplasm, where it is broken down by ATP citrate lyase into oxaloacetate and acetyl-CoA used in fatty acid biosynthesis. ATP citrate lyase activity can be monitored based on the conversion of 5×^13^C-citrate to 2×^13^C-acetyl-CoA and 3×^13^C-oxaloacetate (measured as 3×^13^C-malate). As shown in Figure 8A, 3×^13^C-malate is produced similarly in proliferating, CI7, and CI14 cells, consistent with fibroblasts in all of these states being actively engaged in fatty acid biosynthesis. To more directly assess fatty acid biosynthesis in proliferating and quiescent fibroblasts, we extracted lipids from proliferating, CI7, CI14, and CI14SS7 fibroblasts fed [U-^14^C]-glutamine. The contribution of carbons to fatty acids from glutamine was significantly higher in all of the quiescent fibroblasts compared with the proliferating fibroblasts (Figure 8B), consistent with higher “backwards” flux from α-ketoglutarate to citrate (Figures 3 and S4 and Table S1). The higher levels of fatty acid synthesis in contact-inhibited fibroblasts may contribute to the maintenance of membrane integrity, and may also provide a major sink for cytosolic NADPH.

**Figure 9:**
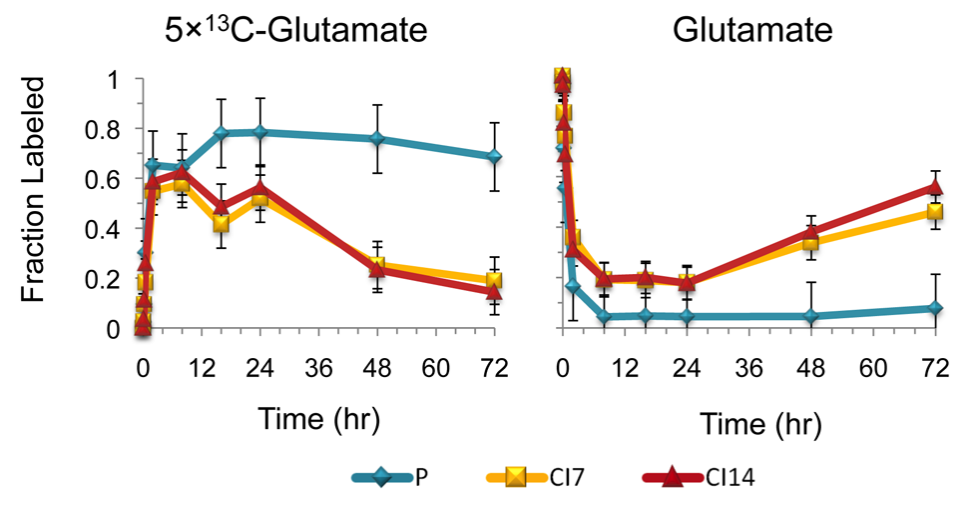
Labeled glutamate levels decrease with time after switching into [U-^13^C]-glutamine in CI7 and CI14 but not proliferating fibroblasts. Proliferating (P), CI7, or CI14 fibroblasts were switched from unlabeled medium to medium containing [U-^13^C]-glutamine, and the fraction of fully labeled glutamate (left plot) and unlabeled glutamate (right plot) was determined over time. Results are an average of four experiments, and error bars indicate standard deviations.

Similarly, our results suggest that contact-inhibited fibroblasts may also be actively degrading existing protein, and thus resynthesizing protein to replace the degraded proteins. As shown in Figure 9, the fraction of glutamate that is labeled in fibroblasts under all conditions increases rapidly after switching cells into [U-^13^C]-glutamine and then drops off in CI7 and CI14 fibroblasts, but not in proliferating fibroblasts. This decline in the fraction of glutamate molecules with five labeled carbons corresponds to an increase in the fraction of unlabeled glutamate. One possible explanation for these data is a breakdown of unlabeled proteins and release of free amino acids into the glutamate pool. These results are in agreement with our quantitative flux analysis: protein synthesis rates are similar across all conditions [43],[44] (Figures 3 and S4 and Table S1). Protein synthesis rates in the best-fit model are 3.3 nmol/min/µg protein for proliferating fibroblasts, 4.3 nmol/min/µg protein for CI7 fibroblasts, 4.1 nmol/min/µg protein for CI14 fibroblasts, and 2.9 nmol/min/µg protein for CI14SS7 fibroblasts. Thus, one reason for the active metabolism observed in contact-inhibited fibroblasts may be to rebuild and thus refresh their lipid and protein contents.

### Contact-Inhibited Fibroblasts Secrete Large Amounts of Extracellular Matrix Proteins

The high metabolic activity of quiescent fibroblasts might also be partially explained by their synthesis and secretion of extracellular matrix molecules needed for the structural integrity of tissue. While proliferating fibroblasts would be expected to secrete molecules important for wound healing [15], quiescent fibroblasts might be expected to secrete extracellular matrix molecules required at the end of a wound healing process or for maintenance of quiescent tissue [45]. We monitored the levels of secreted protein in conditioned medium collected from plates containing proliferating or CI14 fibroblasts [13]. Because serum interferes with immunoblotting for specific proteins, these experiments were performed in no serum and 0.1% serum conditions. As shown in Figure 9, the levels of fibronectin, collagen 21A1, and laminin alpha 2 in conditioned medium from CI14 fibroblasts was higher than the levels in conditioned medium from proliferating fibroblasts, thus demonstrating a biosynthetic commitment for contact-inhibited fibroblasts that may contribute to their high metabolic rate.

### Overview of the Metabolic Changes between Proliferation and Quiescence in Fibroblasts

The metabolic profiles of proliferating and CI14 fibroblasts are summarized in Figure 3. Fibroblasts in both proliferating and contact-inhibited states utilize glycolysis extensively. Proliferating fibroblasts rely on the PPP to generate ribose for nucleotide biosynthesis and NADPH for biosynthetic purposes. Contact-inhibited fibroblasts employ the oxidative PPP to generate NADPH, and the carbon skeletons are largely returned to glycolysis as glyceraldehyde-3-phosphate and fructose-6-phosphate. Fibroblasts in both proliferating and contact-inhibited states contribute some glucose carbons to the TCA cycle. In contact-inhibited fibroblasts, carbons contributed by glucose are transmitted through the TCA cycle; in proliferating fibroblasts, there is little forward flux between citrate and α-ketoglutarate. Contact-inhibited fibroblasts rely more heavily on anaplerotic flux from pyruvate to oxaloacetate via pyruvate carboxylase; proliferating fibroblasts rely more heavily on glutamine, perhaps because of their higher demand for nitrogen. Glutamine drives the forward flux through the TCA cycle and also reverse flux from α-ketoglutarate to citrate, especially in the contact-inhibited fibroblasts. This reverse flux provides a mechanism for shuttling NADPH from mitochondria to the cytosol.

## Discussion

### What Do Quiescent Fibroblasts Do with All of Their Energy?

We discovered that fibroblasts induced into quiescence by contact inhibition maintain a high metabolic rate. In contact-inhibited fibroblasts, nucleotide biosynthesis is reduced, yet the rate of glycolytic, PPP, and TCA flux is almost completely maintained. Even fibroblasts that have been contact-inhibited for 2 wk and starved of serum for the final week show only a 2-fold reduction in glycolytic flux. Contact-inhibited fibroblasts also presumably generate substantial energy through the TCA cycle, where we observed flux of both glucose- and glutamine-derived carbons through more than a complete cycle. Consistent with these multiple routes of energy generation, the ATP/AMP ratio is high in contact-inhibited fibroblasts (Figure S2).

What then do the quiescent fibroblasts do with all of their energy? Our data suggest three avenues for energy utilization. First, contact-inhibited fibroblasts may continuously degrade and resynthesize their macromolecules and membrane components via increased autophagy [43],[44] (E. M. Haley, A. L.-M., and H. A. C., unpublished observation), a strategy that would help to ensure that old and potentially damaged macromolecules and membranes do not accumulate. Our data suggest that contact-inhibited fibroblasts may degrade protein and fatty acids at an enhanced rate compared with proliferating fibroblasts. The conclusion most consistent with our data is that the proliferating and contact-inhibited fibroblasts synthesize amino acids and fatty acids at rates that are comparable, with the new biomass contributing to new cells in proliferating fibroblasts and the new biomass replacing degraded molecules in the contact-inhibited fibroblasts.

Second, contact-inhibited and serum-starved fibroblasts induce pathways that generate NADPH. We discovered that three NADPH-generating enzymes, G6PD, PGD, and IDH1, are expressed at higher levels in quiescent than in proliferating fibroblasts. The results suggest that quiescent fibroblasts activate an NADPH-generating program of enzyme induction. One role of the NADPH may be to ensure the availability of GSH and thioredoxin for the detoxification of free radicals. Indeed, levels of total free radicals are lower in the contact-inhibited than in proliferating fibroblasts (E. M. Haley and H. A. C., unpublished data). Another role for the NADPH generated may be to support resynthesis of fatty acids, as fatty acid degradation yields NADH while synthesis requires NADPH.

**Figure 10:**
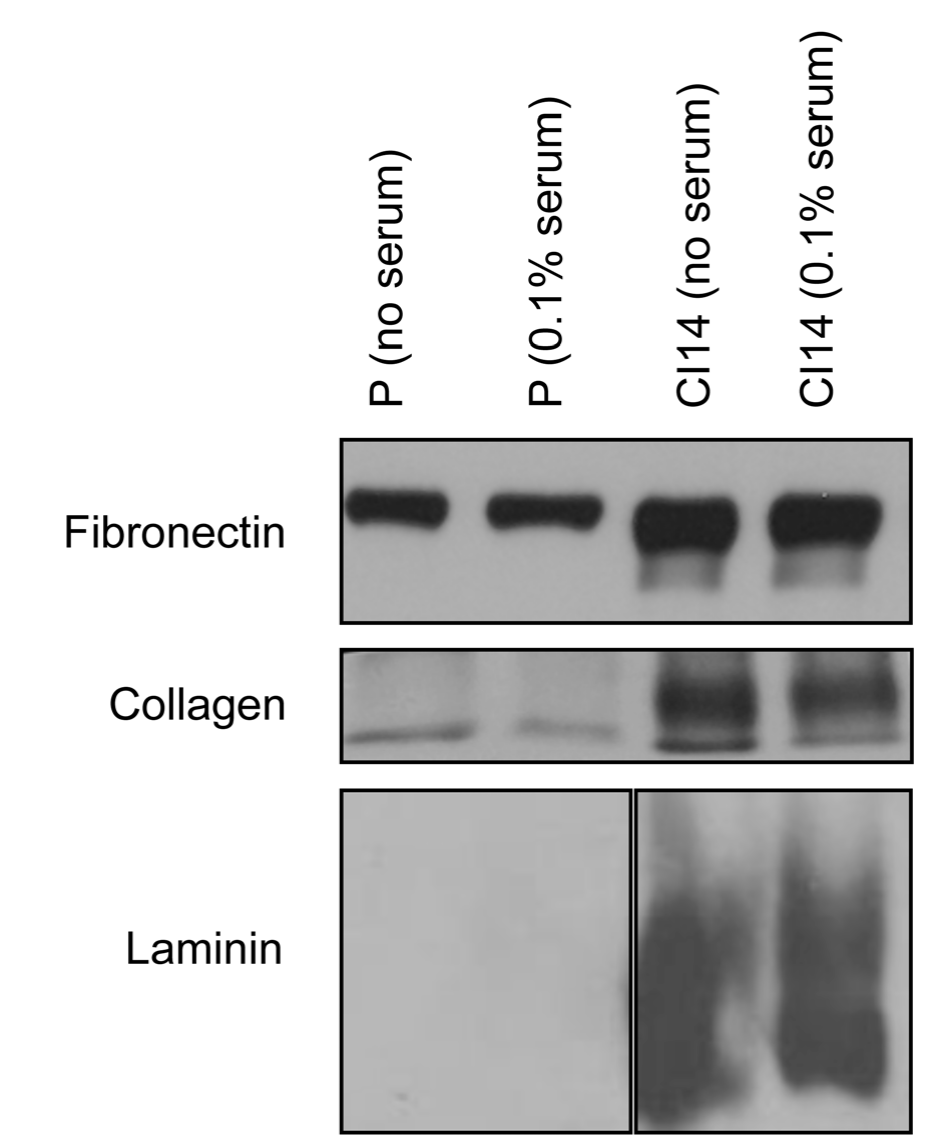
Contact-inhibited fibroblasts secrete high levels of specific extracellular matrix proteins. Conditioned medium (4 d) was collected from proliferating (P) and CI14 fibroblasts conditioned with either no serum or 0.1% serum, and with 0.03% platelet-derived growth factor (PDGF-BB) for proliferating cells. The amount of conditioned medium was normalized to the change in protein content over time. Conditioned medium was precipitated and immunoblotted with an antibody to fibronectin, collagen (col21a1), or laminin (lama2).

Third, quiescent fibroblasts may acquire new cell-type-specific functions. In contrast to lymphocytes, which, with the exception of plasma cells, lack a major biosynthetic function in their quiescent state, fibroblasts secrete proteins and other molecules needed for the extracellular matrix even when they are quiescent. Contact-inhibited fibroblasts direct some of their metabolic activity toward this biosynthetic purpose, as we observed elevated levels of specific extracellular matrix proteins in contact-inhibited compared with proliferating fibroblasts (Figure 10). Thus, quiescent fibroblasts, relieved of the biosynthetic requirements associated with creating progeny, can turn their protein synthesis machinery toward the synthesis of proteins that are beneficial for the organism as a whole.

### What Is Distinctive about Quiescence?

Our findings shed light on some larger questions about quiescence: What are the fundamental attributes of a quiescent state? Is there a single quiescent state or are there multiple quiescent states? Our results suggest that quiescence is not necessarily associated with a shutdown of glycolysis, as reported for lymphocytes and thymocytes [6]–[10]. Quiescent cells can actually be highly metabolically active. In this respect, quiescent fibroblasts resemble terminally differentiated cells like cardiomyocytes, neurons, and renal tubular epithelial cells, which are among the highest energy consumers in mammals. These terminally differentiated cells are well-known to employ nutrients to achieve their contractile, signaling, and transport functions. Whether their metabolic activity, like that of contact-inhibited fibroblasts, is also directed to continuously refreshing their protein and lipid composition merits further study.

In addition to differing from quiescent lymphocytes, different types of quiescent fibroblasts can vary. While CI7, CI14, and CI14SS7 fibroblasts are indistinguishable morphologically or by traditional cell cycle analysis, they differ with regard to their metabolic profiles. Compared with fibroblasts induced into quiescence by contact inhibition, fibroblasts also deprived of serum exhibited a decrease in lactate excretion rates, smaller pool sizes of glycolytic intermediates, and decreased flux from pyruvate to acetyl-CoA. Our findings suggest that cells of different types may actually be in distinct quiescent states, and may have discovered distinct solutions to the metabolic challenges associated with quiescence.

Finally, our findings suggest that contact-inhibited and serum-starved fibroblasts are particularly susceptible to apoptosis induced by treatment with DHEA, a pentose phosphate pathway inhibitor. The ability to selectively kill quiescent cells could have therapeutic potential [46],[47]. For instance, tumor stem cells may exist in a quiescent state for years, while retaining the capacity to emerge from dormancy, proliferate, and initiate a tumor recurrence. Small molecules that target the pathways invoked by these cells to facilitate their survival during dormancy could be useful additions to our therapeutic arsenal. We discovered that contact-inhibited and serum-starved fibroblasts rely on the PPP and possibly other NADPH-generating reactions for viability. Small molecule inhibitors like DHEA might ultimately prove valuable for targeting quiescent tumor cells.

## Materials and Methods

### Tissue Culture

Primary human fibroblasts were isolated from foreskin as previously described (see Supplemental Data in [48]). Fibroblasts were maintained in DMEM (Hyclone, Thermo Fisher Scientific) supplemented with 10% fetal bovine serum (Hyclone) and 100 µg/ml penicillin and streptomycin (Invitrogen). Cells were collected while proliferating, after 1 wk of confluent maintenance (CI7), after 2 wk of confluent maintenance (CI14), after 2 wk of maintenance with the last 7 d in 0.1% serum (CI14SS7), and after serum starvation in 0.1% serum for 3 d, 4 d (SS4), or 7 d (SS7). Cells made quiescent by serum starvation alone were plated sufficiently sparsely so that they did not contact surrounding cells. Medium was changed every 2 d. Proliferating cells were sampled the day after seeding. In order to better simulate conditions in vivo, we also used low glucose/low glutamine conditions in which the glucose level was 1 g/l and the glutamine level was 0.7 mM, compared with a glucose level of 4.5 g/l and a glutamine level of 4 mM in standard DMEM. While cells were confluent, the medium was changed regularly. For analysis, cells were transferred to DMEM (Invitrogen) with 7.5% dialyzed fetal bovine serum (Atlanta Biologicals or Hyclone) the day before the experiment. Fibroblasts were photographed through a Nikon Eclipse TS100 microscope using a Scion 8-bit color firewire 1394 digital camera. Images were captured with Scion VisiCapture software (Scion).

### Flow Cytometry for Cell Cycle

Cells were trypsinized and collected into phosphate-buffered saline (PBS) containing 5% bovine growth serum (Hyclone). Cells were pelleted, resuspended in 67% ethanol in PBS, and stored at 4°C. For flow cytometry, cells were pelleted, washed with PBS, and resuspended in PBS with PI (40 µg/ml) (VWR) and RNAse A (200 µg/ml) (Thermo Fisher Scientific). Samples were incubated in the dark for 1 h at room temperature, and analyzed using a FACSort flow cytometer (BD Biosciences). The PI was excited at 488 nm, and emitted fluorescence was collected on detector FL2 with a bandpass filter of 585/42 nm. At least 20,000 cells were collected and analyzed with CellQuest software (BD Biosciences). Cell cycle distributions were calculated with ModFit LT software using the Watson Pragmatics algorithm.

### Flow Cytometry Analysis for Pyronin Y

To differentiate cells in G_0_ versus G_1_, fibroblasts representing each quiescence condition were trypsinized and suspended in cold Hank's buffered saline solution at a concentration of 2×10^6^ cells/ml, then added to a fixative of ice-cold 70% ethanol. Cells were fixed for at least 2 h, washed, and resuspended at 4×10^6^ cells/ml. A solution of 4 µg/ml pyronin Y and 2 µg/ml Hoechst 33342 was added to the cell suspension and incubated on ice for 20 min before measuring cell cycle status by flow cytometry. To determine RNA content, pyronin Y was excited at 488 nm and emission was measured at 562–588 nm. DNA content was determined by Hoechst 33342. Excitation was measured at 355 nm and emission was measured at 425–475 nm. Cells in G_0_ were identified as the population with 2N DNA content and an RNA content lower than the level in S phase [49].

### Protein Content and Immunoblot Analysis of Proliferating and Quiescent Fibroblasts for p27^Kip1^, IDH1, G6PD, and PGD Levels

Cells were made quiescent by contact inhibition, serum starvation, or a combination as indicated in the text or figure, and collected at the indicated times. The cells were lysed in RIPA buffer (50 mM Tris-Cl [pH 7.4], 150 mM NaCl, 1% Triton X-100, 1% sodium deoxycholate, and 0.1% SDS) containing protease and phosphatase inhibitors (10 mM NaPO4 [pH 7.2], 0.3 M NaCl, 0.1% SDS, 1% NP40, 1% Na deooxycholate, 2 mM EDTA, protease inhibitor cocktail [Roche, Basel, Switzerland] and Halt Phosphatase inhibitors [Thermo Fisher Scientific]). Lysates were sonicated with five pulses for 15 s each at 60 J/W. Lysates were then incubated for 30 min on ice with periodic vortexing and cleared by centrifugation for 2–5 min at 4°C at 10,000 rpm. Total protein amount was assessed by the Lowry method using the Bio-Rad DC Protein Assay Kit II (Bio-Rad) as described by the manufacturer. Spectrophotometer readings taken at 650 nm were compared against a standard curve to determine lysate concentration. Total protein content was determined as the product of lysate concentration and lysate volume. Equal amounts of total cellular proteins were resolved on 12% SDS-PAGE and electro-transferred onto a PVDF membrane. Membranes were blocked for 1 h at room temperature in Tris-buffered saline containing 0.1% Tween-20 (TBS-T) (10 mM Tris [pH 7.6], 15 mM NaCl, and 0.1% Tween-20) or phosphate-buffered saline containing 0.1% Tween-20 (PBS-T) containing 5% nonfat dried milk. Membranes were incubated with antibodies to p27 (1∶500 diluted in TBS-T/5% milk) (Santa Cruz Biotechnology), IDH1 (1 µg/ml diluted in PBS-T/1% milk) (Lifespan Biosciences), G6PD (1∶1,500 diluted in PBS-T/1% milk) (Novus Biologicals), or PGD (1∶1,000 diluted in PBS-T/1% milk) (GeneTex) overnight. Following incubation, the membranes were washed three times in TBS-T or PBS-T and incubated for 1 h with horseradish peroxidase–conjugated anti-rabbit secondary antibody (1∶3,000 diluted into TBS-T/5% milk for p27 or 1∶10,000 diluted in PBS-T/1% milk for IDH1 and G6PD) (GE Healthcare). The membranes were washed three times with TBS-T or PBS-T, and immunoreactive bands were detected with an enhanced chemiluminescence kit (Pierce, Thermo Scientific). The membranes were stripped using Restore Western Blot Stripping Buffer (Thermo Scientific) according to the manufacturer's instructions and immunoblotted with GAPDH (Abcam) (1∶5,000 dilution) in PBS-T/1% milk or TBS-T/5% milk as a loading control.

### Intracellular Metabolite Analysis

Highly parallel measurement of intracellular metabolites was performed as previously described [21]. Metabolites were extracted from proliferating, CI7, CI14, or CI14SS7 cells by aspirating the medium from the plate and flash-quenching metabolic activity with 80% methanol maintained at −80°C. Cells were incubated in methanol for 15 min, scraped on dry ice, and pelleted with centrifugation at 4,400 rpm for 5 min. Samples were re-extracted twice with 80% methanol on dry ice. The three extractions were pooled and dried under nitrogen gas, dissolved in 300 µl of 50% methanol, and spun at 13,000× g for 5 min. Methanol supernatant was then passed through an aminopropyl column [50]. Eluate from the column was analyzed with positive ion mass spectrometry via a Finnigan TXQ Quantum Ultra triple-quadrupole mass spectrometer equipped with an electrospray ionization source (Thermo Fisher Scientific) [22]. A TSQ Quantum Discovery MAX mass spectrometer, also equipped with an electrospray ionization source, was used to collect data on negative mode ions after separation on a 25-cm C18 column coupled with a tributylamine ion pairing agent to aid in the retention of polar compounds [51],[52].

To quantify metabolites, peak heights were initially assigned using XCalibur software (Thermo Fisher Scientific) and then evaluated manually. Metabolites enriched at least 5-fold in a sample compared with a control plate containing only medium were retained in the analysis. Of the 172 metabolites monitored, 62 met these criteria. Signals that were below the limit of detection were assigned 100. Metabolite levels were normalized by the amount of protein present.

### Metabolic Flux Analysis

To monitor the flux through metabolic pathways, samples were incubated with medium containing isotope-labeled nutrient for different amounts of time. Dulbecco's medium lacking glucose and glutamine was isotope-labeled by adding back glucose or glutamine ([U-^13^C]-glucose, [1, 2-^13^C]-glucose, [3-^13^C]-glucose, or [U-^13^C]-glutamine; Cambridge Isotope Laboratories) to a final concentration of 4.5 g/l glucose or 0.584 g/l glutamine. Samples were taken at the indicated time points after medium change and processed as described above. Levels of ^12^C and ^13^C forms of metabolic intermediates were monitored with LC-MS/MS [53].

### Metabolite Uptake and Excretion

Medium was sampled from cells under a variety of conditions: proliferating, CI7, CI14, CI14SS7, SS4, SS7, low glucose/low glutamine proliferating, and low glucose/low glutamine CI14. Conditioned medium was sampled over a time course from 0 to 96 h for fibroblasts, depending upon the experiment. The levels of glucose, lactate, glutamine, and glutamate were measured using a YSI 7100 Select Biochemistry Analyzer (YSI Incorporated). The rate of glucose consumption, lactate excretion, glutamine consumption, and glutamate excretion was determined as the rate that these metabolites appeared or disappeared from the medium divided by the time integral of the protein mass of cells on the plate during that time period.

### Glutathione Measurement

The total GSH and GSSG content of proliferating, CI7, CI14, and CI14SS7 fibroblasts were determined using Cayman Chemical's Glutathione Assay Kit according to the manufacturer's instructions (Cayman Chemical). Cayman's GSH assay kit employs a carefully optimized enzymatic recycling method, using glutathione reductase for the quantification of GSH. Briefly, cells were harvested using a cell lifter in 1.5 ml of cold buffer (i.e., 50 mM MES or phosphate buffer [pH 6–7] containing 1 mM EDTA) and were centrifuged at 10,000× g for 15 min at 4°C, followed by metaphosphoric acid deproteinization and addition of triethanolamine solution. Half of the samples were then treated with 2-vinylpyridine to allow quantification of the GSSG pool exclusively. Assay Cocktail (a mixture of 2-(N-morpholino) ethanesulfonic acid Buffer [11.25 ml], reconstituted Cofactor Mixture [0.45 ml], reconstituted Enzyme Mixture [2.1 ml], water [2.3 ml], and reconstituted 5,5′-dithiobis-(2-nitrobenzoic acid) [0.45 ml]) was added, and total GSH and GSSG in the deproteinated samples were measured at 405 nm in a spectrophotometer. GSH concentration of the samples was determined by the endpoint method and expressed in micromolar concentrations.

### PPP Inhibition and PI Live/Dead Analysis

Proliferating and CI14 fibroblasts were treated with DHEA dissolved in ethanol or dimethylsufoxide (0.1% vol/vol) for 4 d. On the fourth day of treatment with the inhibitor, cells were trypsinized and collected into conditioned medium. Cells were then centrifuged for 5 min at 1,000 rpm. The supernatant was aspirated and cells were taken up in PBS with 1 µg/ml PI (VWR). Cells were kept on ice and immediately analyzed by flow cytometry using a BD LSRII multi-laser analyzer (BD Biosciences). PI was excited at 488 nm, and emitted fluorescence was collected through a 610/20 bandpass filter. At least 40,000 cells were collected and analyzed with FACSDiVa software (BD Biosciences). PI-negative cells were counted as live cells, and PI-positive cells were counted as dead cells.

### PPP Inhibition and Apoptosis Analysis

Apoptosis was measured based on the levels of caspase-3/7 released into the medium using the ApoTox-Glo Triplex Assay according to the manufacturer's instructions (Promega). Cells were plated in triplicate at 10,000 cells per well in white-walled, clear-bottom 96-well plates (Costar, Corning Life Sciences). For contact inhibition, cells were plated 7 d prior to the start of treatment; for serum starvation, cells were plated 4 d prior to treatment and switched to 0.1% serum medium for the remaining 3 d; proliferating cells were plated the day prior to the start of treatment. Increasing concentrations of DHEA or ethanol vehicle alone were added to the medium in each well, and treatment proceeded for 4 d. Cells in serum starvation conditions were incubated in 0.1% serum during treatment as well. The apoptosis reagent was added at 100 µl per well and incubated for 1 h prior to reading. Luminescence was read from the top using a Synergy-2 plate reader (Biotek). Luminescence data were normalized to the vehicle only condition.

### Measurement of Carbon Incorporation into Fatty Acids

Lipid synthesis from glutamine was measured using a modified version of a previously published protocol [53]. Briefly, proliferating, CI7, CI14, and CI14SS7 fibroblasts were incubated in medium containing 5 µCi/ml [U-^14^C]-glutamine at 4 mM (0.4% labeled). After incubation for 24 h, the culture medium was aspirated, cells were washed with PBS, and phospholipids were extracted by addition of 500 µl of 3∶2 hexane∶isopropanol. The culture dishes were then washed with an additional 500 µl of the hexane∶isopropanol mixture. The resulting total extract was dried using a speed-vac, resuspended in 500 µl of 1 N KOH in 90∶10 methanol∶water, and incubated at 70°C for 60 min to saponify lipids. Sulfuric acid (100 µl, 2.5 M) was then added, followed by hexane (700 µl) to extract the saponified fatty acids. The organic and aqueous phases were separated by centrifugation and scintillation-counted.

### Microarray Analysis

To monitor gene expression levels, proliferating, CI7, or CI14 fibroblasts were trypsinized, removed from the plate, pelleted, and stored at −80°C. Total RNA was isolated using the mirVana miRNA Isolation kit (Ambion) according to the manufacturer's instructions. RNA quality was verified using a Bioanalyzer 2100 (Agilent Technology), and the amount was determined with a NanoDrop spectrophotometer (NanoDrop Technologies). Total RNA (325 ng) was amplified using the Low RNA Input Fluorescent Labeling Kit (Agilent Technologies) according to the manufacturer's protocol. Cy-3 (PerkinElmer) was directly incorporated into the cRNA from proliferating cells during in vitro transcription. Cy-5 was incorporated into complementary RNA from CI7 or CI14 fibroblasts. Mixtures of Cy-3-labeled and Cy-5-labeled cRNA were co-hybridized to Whole Human Genome Oligo Microarray slides (Agilent Technologies) at 60°C for 17 h and subsequently washed according to the Agilent Technologies standard hybridization protocol. Slides were scanned with a dual laser scanner (Agilent Technologies). Images were monitored for quality control. The Agilent Technologies feature extraction software, in conjunction with the Princeton University MicroArray database (http://puma.princeton.edu/), was used to compute the log ratio of the two samples for each gene after background subtraction and dye normalization. The entire experiment was performed twice.

### Analysis of Extracellular Matrix Protein Levels in Conditioned Medium

For the analysis of extracellular matrix proteins in conditioned medium, we could not perform the experiments in the presence of high amounts of serum because serum inhibited protein transfer after immunoblotting. As previously described [13], proliferating fibroblasts were conditioned at low cell density in the presence of platelet-derived growth factor with either no serum or 0.1% serum. Quiescent fibroblasts were cultured at high density in the absence of platelet-derived growth factor with either no serum or 0.1% serum. Medium was conditioned over 4 d and during that time, protein lysates were collected over a time course. The protein content of the cell lysates was plotted against the time of lysate collection. A curve that fit the data was generated and the area under the curve, the integrated protein–hour quantity, was divided by the volume of medium collected from the proliferating or quiescent plate. The total protein–hour/volume for each sample was used to adjust the volume of conditioned medium, which was then mixed with 25% volume of trichloroacetic acid (Sigma-Aldrich) containing 0.1% sodium deoxycholate (Sigma-Aldrich), and incubated for 30 min on ice. Following centrifugation, samples were washed 3–4 times with −20°C acetone, resuspended in sodium dodecyl sulfate-polyacrylamide gel electrophoresis sample buffer and separated under reducing conditions on 5% (for fibronectin and COL21A1) or 12% (for LAMA2) sodium dodecyl sulfate-polyacrylamide gels. Proteins were transferred for 1 h at 100 V to Westran polyvinylidene fluoride membranes (PerkinElmer). Membranes were blocked for 1 h at room temperature in 5% nonfat dried milk in PBS-T. Membranes were then incubated overnight at 4°C with a mouse monoclonal anti-fibronectin clone HFN7.1 (1∶2,000 dilution, generous gift of Jean Schwarzbauer, Princeton University), mouse polyclonal antibody against COL21A1 (1∶750 dilution, Abcam), or mouse monoclonal antibody against LAMA2 (3 µg/ml, Abnova) diluted in PBS-T/1% milk. Following overnight incubation in the primary antibody, membranes were washed three times in PBS-T, incubated for 1 h in a 1∶10,000 dilution of horseradish peroxidase–conjugated sheep anti-mouse secondary antibody (GE Healthcare) in PBS-T/1% milk. Membranes were exposed to X-ray film, and film was scanned with a Hewlett-Packard Scanjet 4890 using Hewlett-Packard software. The intensity of individual bands was determined with ImageJ analysis software.

### Computational Determination of Fluxes

Fluxes were determined by integration of all available forms of experimental data within a quantitative flux-balanced framework using the same strategy as described in Munger et al. [53]. An ODE model (Figure S3) of central carbon metabolism was constructed. The model assumes steady-state, mass-balanced flux and simulates the resulting labeling dynamics after switching cells from unlabeled medium to uniformly ^13^C-labeled glucose or glutamine. The model consists of 55 ODEs, describing the rate of loss of unlabeled metabolites and the rate of accumulation of labeled metabolites. It builds upon the previously described model [53] with a few changes. An exchange flux (*F*_12_) was introduced in glycolysis between DHAP and FBP. Backward flux (*F*_11_) from α-ketoglutarate to citrate, together with a latent citrate pool that is never labeled (determined by the lowest unlabeled citrate pool size observed in all experiments), was introduced in the TCA cycle. The latent citrate pool was added because for citrate, but not other metabolites, a substantial fraction of the pool (approximately 40% for the proliferating cells) did not label over the course of the experiment. Beyond labeling dynamics, additional input data included metabolite levels, rates of metabolite consumption and excretion, and the glycolysis–PPP flux convergence ratio determined after feeding [1, 2-^13^C]-glucose for 2 h. Model parameters (fluxes, as well as pool sizes of a small number of metabolites that could not be directly experimentally measured) were identified by a genetic algorithm that minimizes a cost function defined as the sum of weighted differences between the experimental data and computational results (Table S3) [54]. As a global search algorithm, the genetic algorithm computationally probes for alternative flux solutions consistent with the experimental results. For each cell type, the algorithm was run until 1,000 consistent solutions (i.e., parameter sets that produced the lowest cost values when the algorithm reached convergence) were obtained. The distribution of the 1,000 values was then used to quantitatively represent each identified parameter. Since the distributions are not Gaussian, a flux is considered quantitatively different between proliferating and quiescent cells only when the distributions from the proliferating and quiescent fibroblasts do not overlap. This measure minimizes the false positives that may occur when only one or a few solutions are identified [54]. Although qualitatively supportive of the model-inferred enhancement of anapleurotic flux from glucose in quiescent fibroblasts, labeling data for [3-^13^C]-glucose, which was taken at 8 h, were quantitatively inconsistent with the other labeling data, which covered the first 2 h of incubation only. The [3-^13^C]-glucose data were accordingly excluded from the computational analysis. The computer code is available upon request.

### Accession Numbers

The Entrez Gene (http://www.ncbi.nlm.nih.gov/gene) accession numbers for the proteins discussed in this paper are G6PD, 2539; IDH1, 3417; IDH2, 3418; and PGD, 5226.

## Supporting Information

Figure S1 **Glycolytic rates in proliferating and quiescent fibroblasts.** (A) Rates of glucose consumption, lactate excretion, glutamine consumption, and glutamate excretion were monitored in proliferating, CI7, CI14, CI14SS7, SS4, SS7, proliferating low glucose/low glutamine, and CI14 low glucose/low glutamine fibroblasts using the YSI 7100 bioanalyzer. Levels were normalized for the amount of cellular protein present during the conditioning time. Error bars indicate standard error. (B–E) Representative plots of metabolite levels over time used to determine the reported rates.
(7.08 MB PDF)

Figure S2 **Basal metabolites in proliferating, CI7, and CI14 fibroblasts.** Metabolites were analyzed using LC-MS/MS. Individual metabolite levels were normalized for protein content. The log (base 2) of the ratio of CI7 or CI14 to the average proliferating metabolite levels over all experiments was determined for each sample. Means from four experiments each containing 4–5 replicates are shown. Error bars indicate standard error.
(0.59 MB TIF)

Figure S3 **Flux-balanced model of central carbon metabolism.** An ODE-based model of central carbon metabolism was developed to describe the time-dependent metabolic labeling. (A) Schematic of fluxes in the model. *F*_0_–*F*_12_ represent the unknown fluxes, except for *F*_9_, which is the latent hexose–phosphate pool. *A*, *B*, *C*, and *D* are the uptake and excretion rates. *X*, *Y*, and *Z* are dependent parameters of the above fluxes and pool sizes, whose expressions are determined by balancing all the relevant fluxes. *X* is the protein synthesis rate, *Y* is the anaplerotic flux from pyruvate, and *Z* is the net flux from malate to oxaloacetate. (B) Conversion of the isotopically labeled metabolic forms in the glucose and glutamine labeling experiments. The numbers under the metabolite names represent the positions at which a metabolite is labeled (“0” means an unlabeled metabolite). Low-abundance isotope-labeled forms, such as 1×^13^C-citrate, were excluded from the model.
(1.15 MB TIF)

Figure S4 **Modeling results for central carbon metabolism.** (A) Model fits for metabolites in proliferating, CI7, CI14, and CI14SS7 fibroblasts. Experimentally measured concentrations of different labeled and unlabeled metabolites (mean ± one standard deviation) at the indicated time points are plotted against the model predictions (smooth curves) from a typical flux solution set. The time axis is in logarithmic scale to better illustrate the samples at early time points. Data and simulated results for [U-^13^C]-glucose labeling experiments are labeled by the metabolite name only. For the [U-^13^C]-glutamine labeling, a “Q” precedes the metabolite name. (B) Histogram of the distribution of consistent fluxes in each condition. The *x*-axis indicates the flux values (in logarithmic scale); the *y*-axis is the number of counts (within the 1,000 consistent solution sets) that have a specific flux value. The resultant solution distribution provides a representation of the fluxes that are potentially consistent with the observed laboratory data in each cell state.
(0.43 MB TIF)

Table S1 **Absolute fluxes in proliferating and quiescent fibroblasts.** For each identified flux, the median value of its distribution (A) and the best value (i.e., the one that resulted in the best match between the experimental data and computational simulations) (B) are reported. Flux values that are statistically higher in quiescent than proliferating conditions (i.e., their distributions do not overlap) are highlighted in red, while the fluxes that are lower in quiescent than proliferating conditions are highlighted in blue.
(0.07 MB DOC)

Table S2 **Malate and citrate labeling after incubation with [3-^13^C]-glucose in proliferating, CI7, CI14, and CI14SS7 fibroblasts.**
(0.01 MB XLS)

Table S3
**Functional forms of the components of the cost function for the genetic algorithm.** The expression for the total cost is
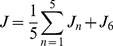
(1)The best possible cost value is 1, when the model results fit all experimental data perfectly.(0.06 MB DOC)

## Abbreviations

CI7: contact-inhibited for 7 d
CI11: contact-inhibited for 11 d
CI14: contact-inhibited for 14 d
CI14SS7: contact-inhibited for 14 d and serum-starved for 7 d
DHAP: dihydroxyacetone phosphate
DHEA: dehydroepiandrosterone
DMEM: Dulbecco's Modified Eagle Medium
FBP: fructose-1,6-bisphosphate
G6PD: glucose-6-phosphate dehydrogenase
GSH: reduced glutathione
GSSG: oxidized glutathione
IDH1: isocitrate dehydrogenase 1
IDH2: isocitrate dehydrogenase 2
LC-MS/MS: liquid chromatography–tandem mass spectrometry
ODE: ordinary differential equation
PBS: phosphate-buffered saline
PBS-T: phosphate-buffered saline containing 0.1% Tween-20
PGD: 6-phosphogluconate dehydrogenase
PI: propidium iodide
PPP: pentose phosphate pathway
SS4: serum-starved for 4 d
SS7: serum-starved for 7 d
TBS-T: tris-buffered saline containing 0.1% Tween-20
TCA: tricarboxylic acid
