# Supplementary figures and images for: Quiescent Fibroblasts Exhibit High Metabolic Activity
Source: PLoS Biol. 2010 Oct 19;8(10):e1000514. doi: 10.1371/journal.pbio.1000514 (PMC2958657; doi:10.1371/journal.pbio.1000514)

**A**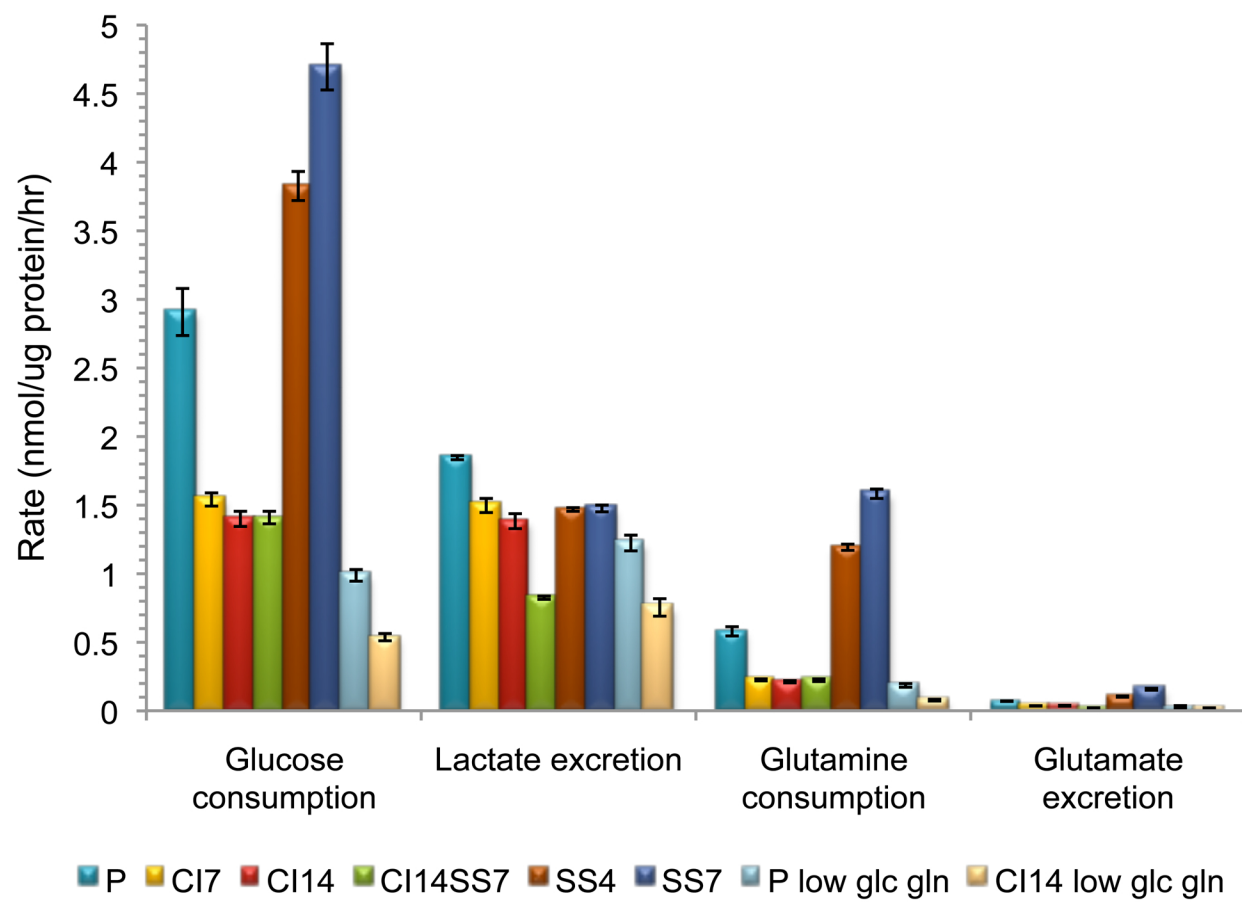

**B**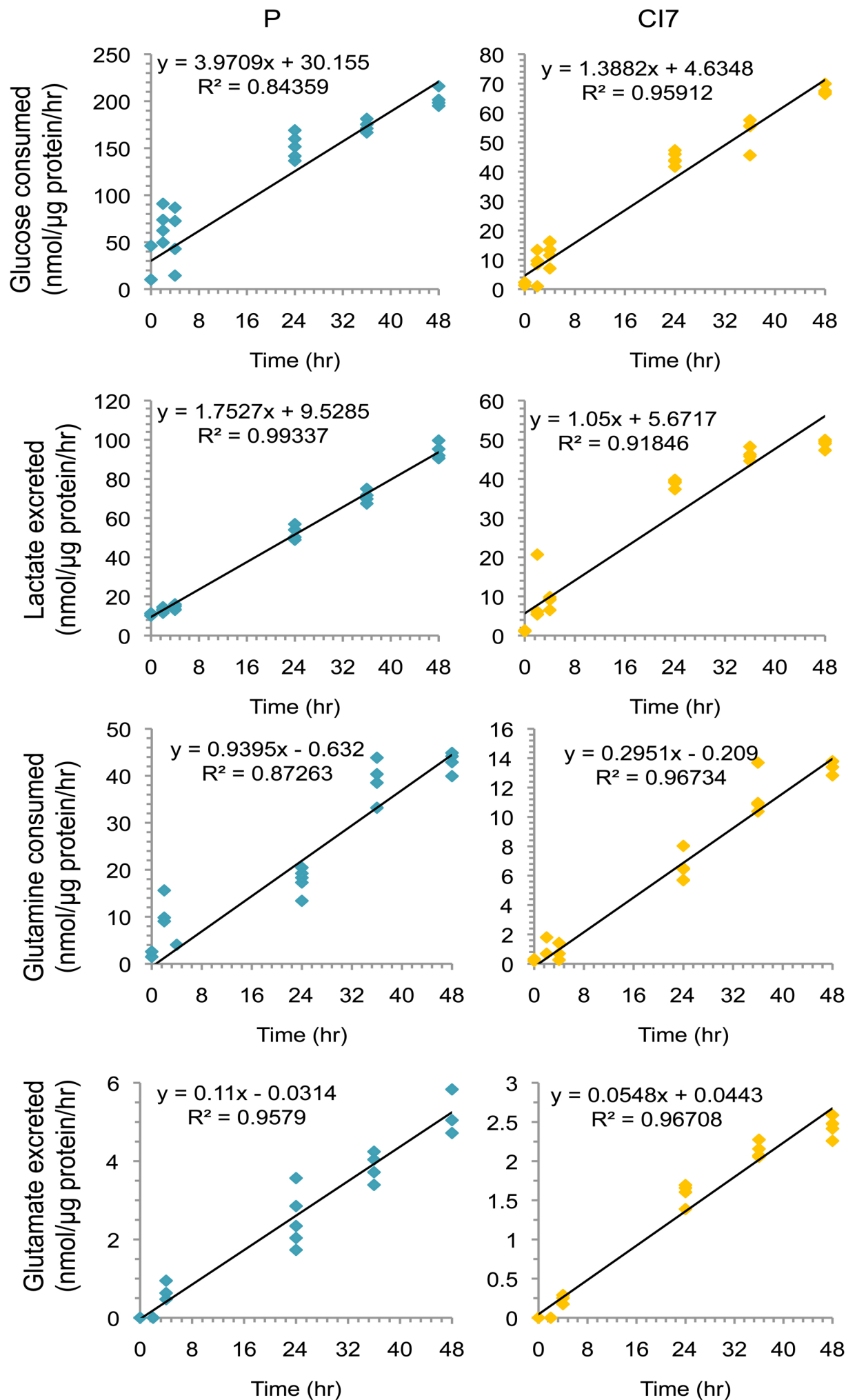

**C**

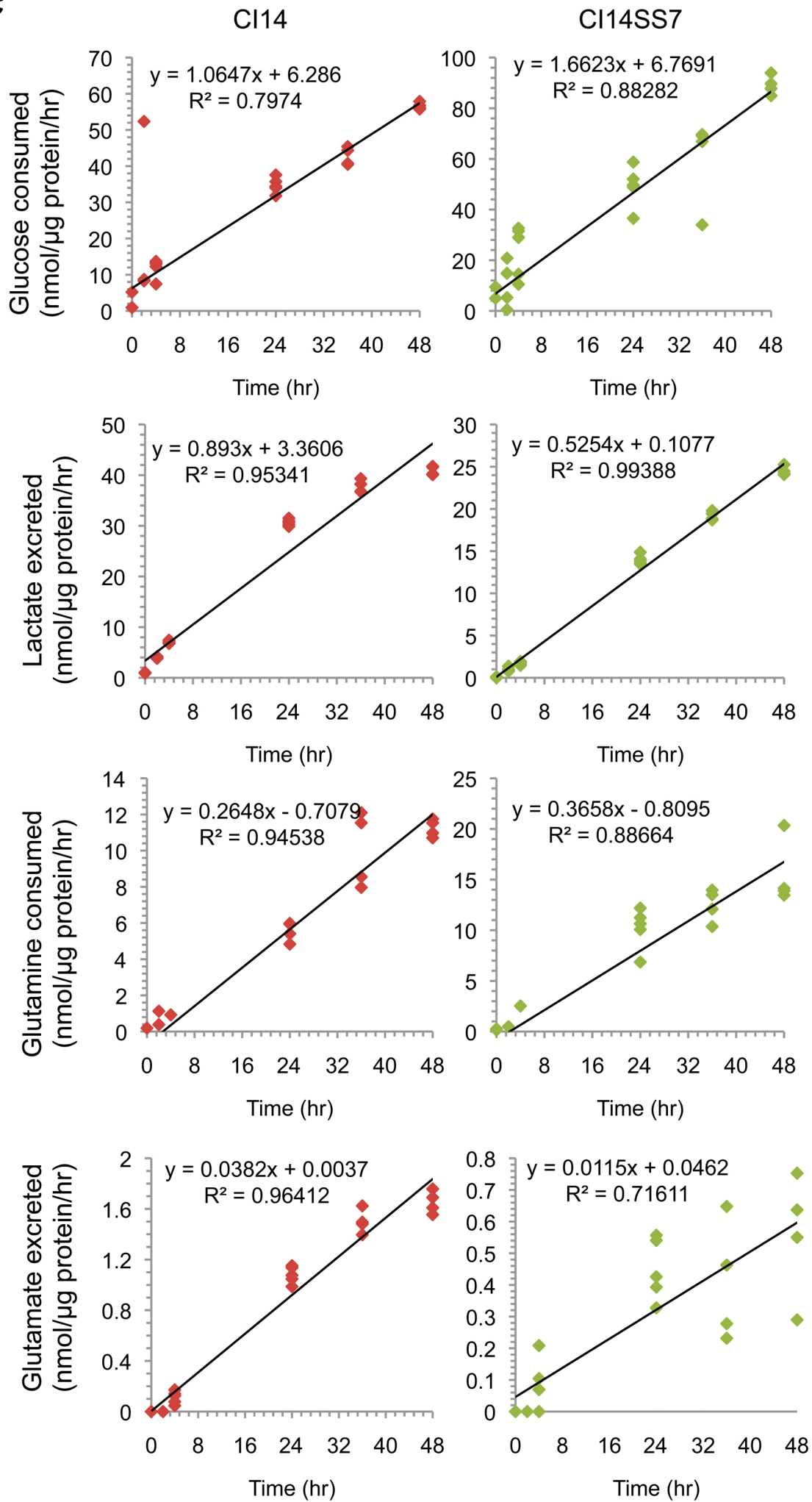

D

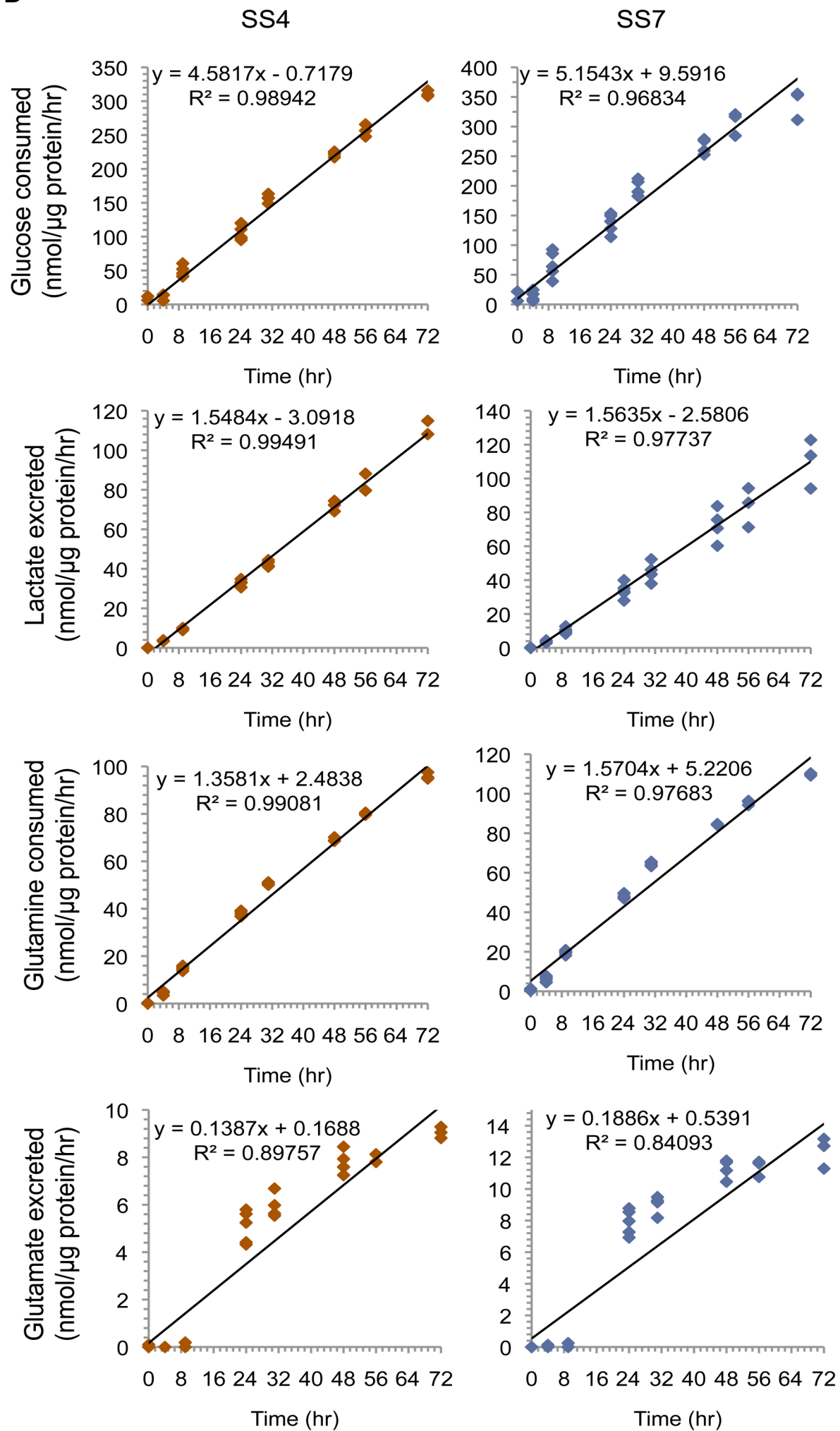

E

P Low Gluc Low Gln

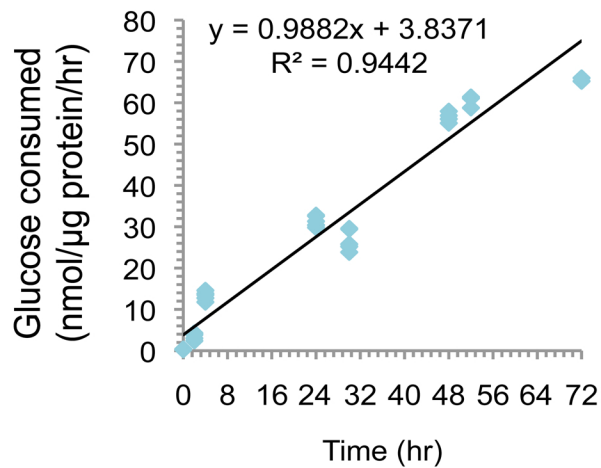

C14 Low Gluc Low Gln

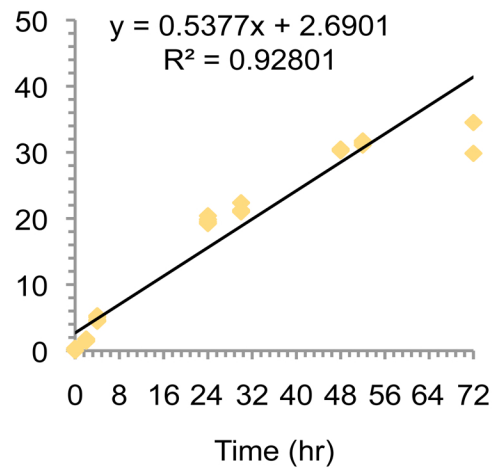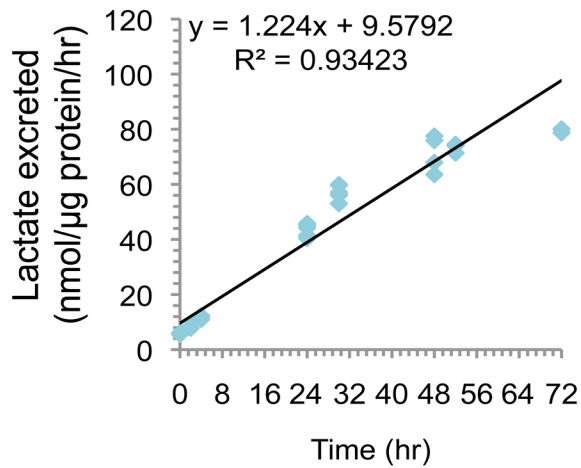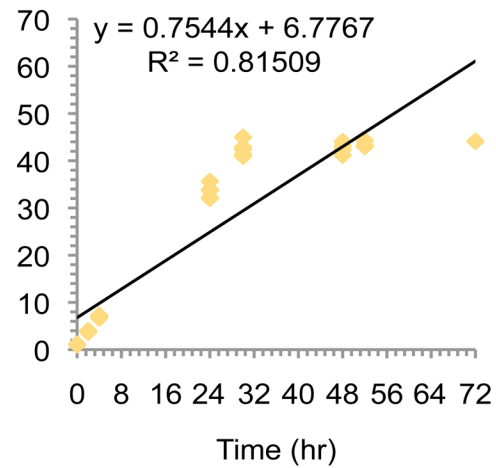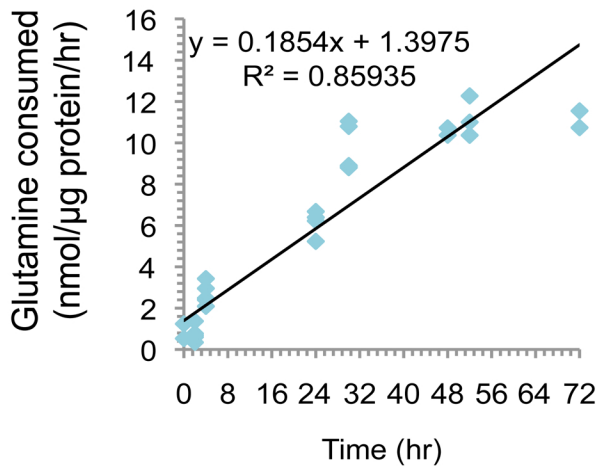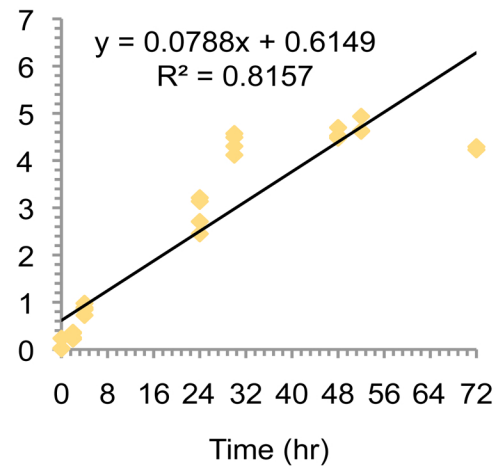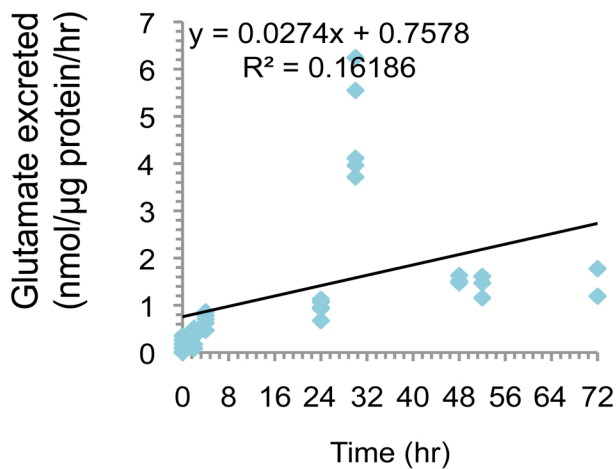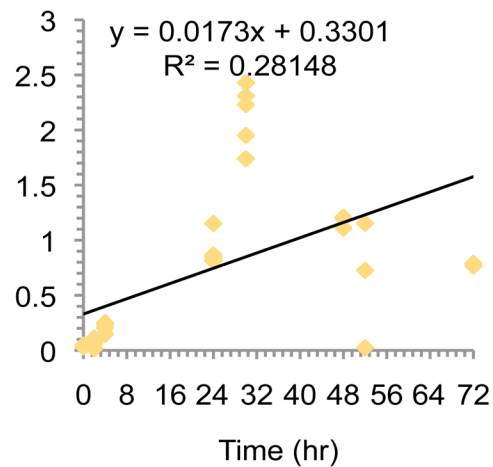

Supplement: Figure S1 — Glycolytic rates in proliferating and quiescent fibroblasts. (A) Rates of glucose consumption, lactate excretion, glutamine consumption, and glutamate excretion were monitored in proliferating, CI7, CI14, CI14SS7, SS4, SS7, proliferating low glucose/low glutamine, and CI14 low glucose/low glutamine fibroblasts using the YSI 7100 bioanalyzer. Levels were normalized for the amount of cellular protein present during the conditioning time. Error bars indicate standard error. (B–E) Representative plots of metabolite levels over time used to determine the reported rates. (7.08 MB PDF) [file pbio.1000514.s001.pdf]

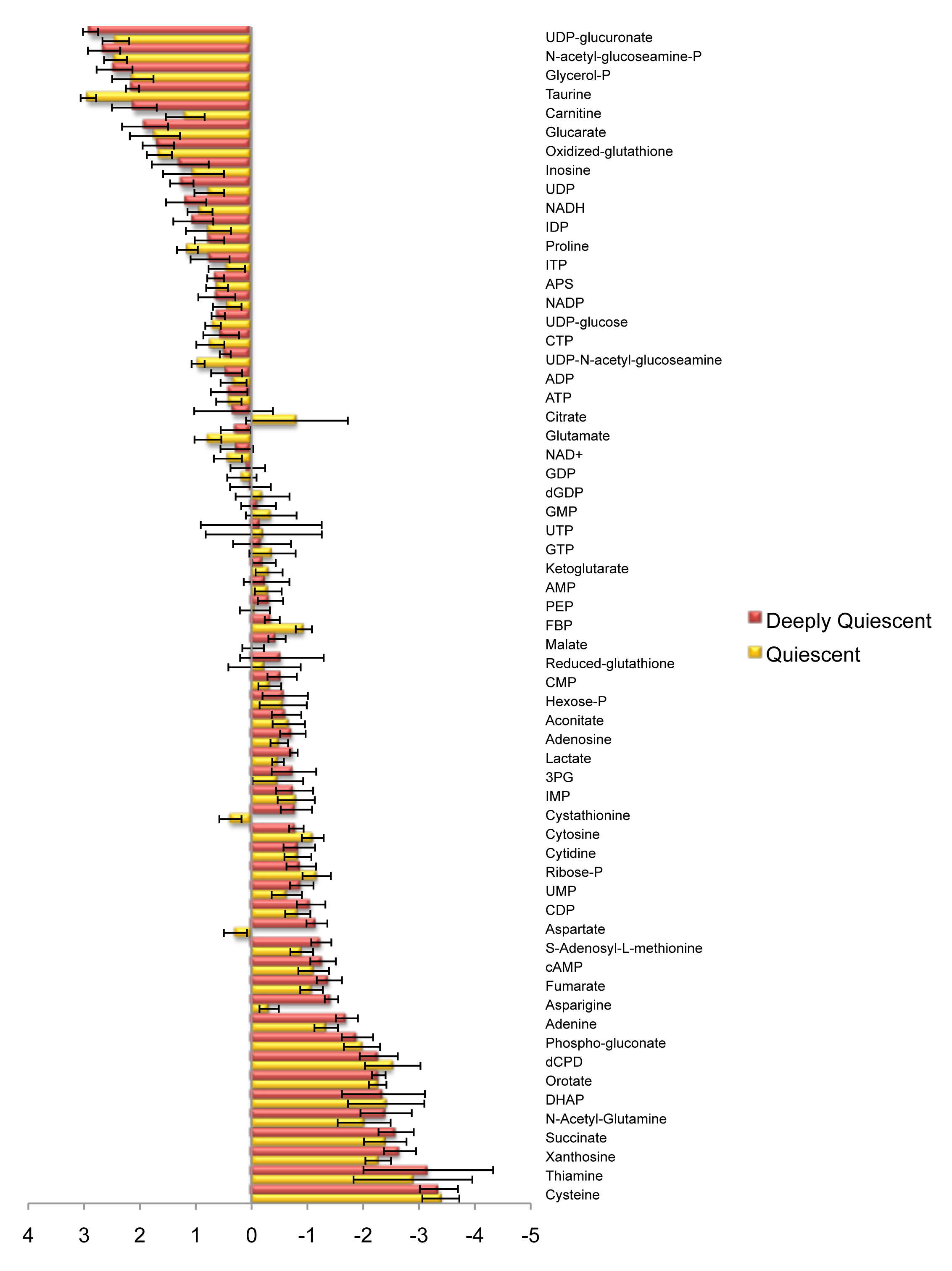

Supplement: Figure S2 — Basal metabolites in proliferating, CI7, and CI14 fibroblasts. Metabolites were analyzed using LC-MS/MS. Individual metabolite levels were normalized for protein content. The log (base 2) of the ratio of CI7 or CI14 to the average proliferating metabolite levels over all experiments was determined for each sample. Means from four experiments each containing 4–5 replicates are shown. Error bars indicate standard error. (0.59 MB TIF) [file pbio.1000514.s002.tif]

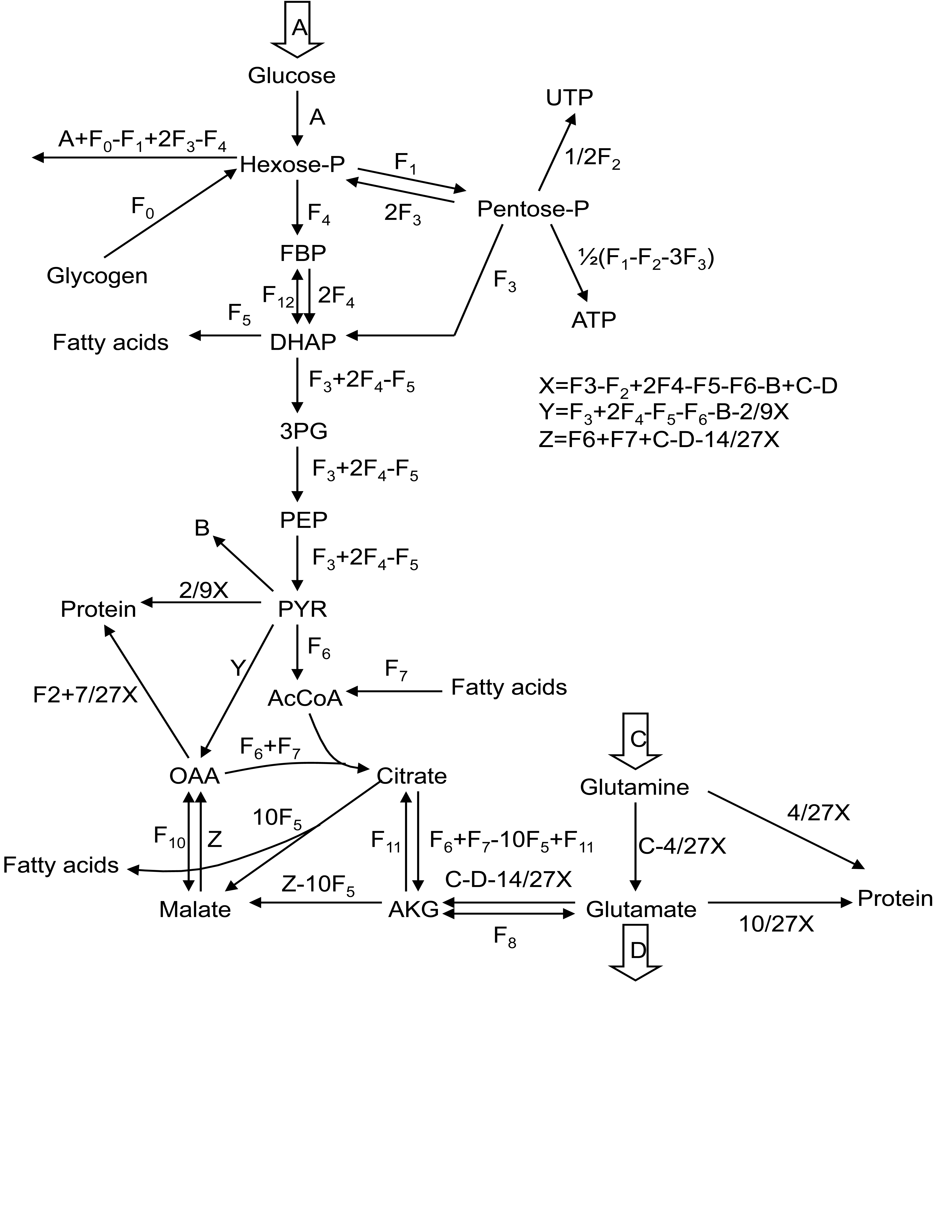

Supplement: Figure S3 — Flux-balanced model of central carbon metabolism. An ODE-based model of central carbon metabolism was developed to describe the time-dependent metabolic labeling. (A) Schematic of fluxes in the model. F 0–F 12 represent the unknown fluxes, except for F 9, which is the latent hexose–phosphate pool. A, B, C, and D are the uptake and excretion rates. X, Y, and Z are dependent parameters of the above fluxes and pool sizes, whose expressions are determined by balancing all the relevant fluxes. X is the protein synthesis rate, Y is the anaplerotic flux from pyruvate, and Z is the net flux from malate to oxaloacetate. (B) Conversion of the isotopically labeled metabolic forms in the glucose and glutamine labeling experiments. The numbers under the metabolite names represent the positions at which a metabolite is labeled (“0” means an unlabeled metabolite). Low-abundance isotope-labeled forms, such as 1×13C-citrate, were excluded from the model. (1.15 MB TIF) [file pbio.1000514.s003.tif]
