# Supplementary material for: Quiescent Fibroblasts Exhibit High Metabolic Activity
Source: PLoS Biol. 2010 Oct 19;8(10):e1000514. doi: 10.1371/journal.pbio.1000514 (PMC2958657; doi:10.1371/journal.pbio.1000514)
Supplement: Table S1 — Absolute fluxes in proliferating and quiescent fibroblasts. For each identified flux, the median value of its distribution (A) and the best value (i.e., the one that resulted in the best match between the experimental data and computational simulations) (B) are reported. Flux values that are statistically higher in quiescent than proliferating conditions (i.e., their distributions do not overlap) are highlighted in red, while the fluxes that are lower in quiescent than proliferating conditions are highlighted in blue. (0.07 MB DOC) [file pbio.1000514.s005.doc]

Supplementary Table S1A: Median values of the absolute fluxes in proliferating and quiescent fibroblasts.

| Cell type | P | CI7 | CI14 | CI14SS7 |
| --- | --- | --- | --- | --- |
| Glycogen to HexP | 1.1 | 0.1 | 0.6 | 2.2 |
| Hex-p to RibP | 1.3 | 1.4 | 1.6 | 0.9 |
| RibP to ATP | 0.4 | 0.2 | 0.1 | 0.2 |
| RibP to UTP | 0.06 | 0.2 | 0.01 | 0.002 |
| RibP to DHAP | 0.2 | 0.3 | 0.4 | 0.1 |
| HexP to FBP | 15.8 | 15.8 | 15.8 | 8.9 |
| FBP-DHAP exchange | 7.9 | 14.1 | 12.6 | 1.7 |
| DHAP to 3PG | 31.8 | 31.9 | 32.0 | 18.0 |
| PYR to AcCoA | 0.6 | 0.4 | 0.2 | 0.003 |
| FA to AcCoA | 0.6 | 0.7 | 2.0 | 0.04 |
| PYR to OAA | 0.02 | 0.06 | 0.1 | 0.08 |
| AcCoA to CIT | 1.2 | 1.1 | 2.2 | 0.06 |
| CIT to AKG | 1.2 | 1.9 | 2.6 | 0.7 |
| AKG to CIT | 0.8 | 1.4 | 1.6 | 0.7 |
| AKG to MAL | 1.1 | 1.5 | 1.7 | 0.4 |
| FA synthesis | 0.8 | 0.6 | 1.3 | 0.1 |
| GLT to AKG | 0.7 | 1.0 | 0.7 | 0.5 |
| GLT-AKG exchange | 200 | 316 | 1122 | 2.2 |
| GLN to GLT | 3.1 | 3.5 | 3.4 | 2.1 |
| MAL to OAA | 1.9 | 2.0 | 2.9 | 0.5 |
| OAA-MAL exchange | 891 | 794 | 794 | 56 |
| GLC uptake | 23.8 | 22.9 | 21.9 | 14.2 |
| LAC excretion | 30.4 | 30.5 | 30.7 | 17.3 |
| GLN uptake | 3.6 | 4.1 | 4.0 | 2.5 |
| GLT excretion | 1.2 | 0.9 | 1.2 | 0.6 |
| Protein synthesis rate | 3.2 | 4.3 | 4.1 | 2.8 |

Supplementary Table S1B: Best values of the absolute fluxes in proliferating and quiescent fibroblasts.

| Cell type | P | CI7 | CI14 | CI14SS7 |
| --- | --- | --- | --- | --- |
| Glycogen to HexP | 1.1 | 0.6 | 0.6 | 2.0 |
| Hex-p to RibP | 1.3 | 1.8 | 1.8 | 1.1 |
| RibP to ATP | 0.4 | 0.2 | 0.1 | 0.3 |
| RibP to UTP | 0.03 | 0.1 | 0.001 | 0.0002 |
| RibP to DHAP | 0.3 | 0.8 | 1.0 | 0.4 |
| HexP to FBP | 15.8 | 17.8 | 15.8 | 8.9 |
| FBP-DHAP exchange | 7.9 | 14.1 | 12.6 | 2.0 |
| DHAP to 3PG | 31.8 | 35.9 | 32.1 | 18.0 |
| PYR to AcCoA | 0.9 | 0.4 | 0.3 | 0.002 |
| FA to AcCoA | 0.7 | 0.8 | 2.0 | 0.14 |
| PYR to OAA | 0.03 | 0.06 | 0.1 | 0.09 |
| AcCoA to CIT | 1.6 | 1.2 | 2.2 | 0.2 |
| CIT to AKG | 1.6 | 2.1 | 2.6 | 0.2 |
| AKG to CIT | 1.0 | 1.4 | 1.6 | 0.2 |
| AKG to MAL | 1.3 | 1.7 | 1.7 | 0.5 |
| FA synthesis | 1.0 | 0.6 | 1.3 | 0.2 |
| GLT to AKG | 0.7 | 1.0 | 0.7 | 0.5 |
| GLT-AKG exchange | 112 | 89 | 562 | 0.4 |
| GLN to GLT | 3.1 | 3.7 | 3.4 | 2.3 |
| MAL to OAA | 2.3 | 2.3 | 2.9 | 0.6 |
| OAA-MAL exchange | 891 | 794 | 891 | 8.9 |
| GLC uptake | 22.2 | 21.9 | 20.9 | 13.0 |
| LAC excretion | 30.1 | 34.4 | 30.8 | 17.0 |
| GLN uptake | 3.6 | 4.3 | 4.0 | 2.7 |
| GLT excretion | 1.2 | 1.1 | 1.2 | 0.7 |
| Protein synthesis rate | 3.3 | 4.3 | 4.1 | 2.9 |
