# Supplementary material for: Quiescent Fibroblasts Exhibit High Metabolic Activity
Source: PLoS Biol. 2010 Oct 19;8(10):e1000514. doi: 10.1371/journal.pbio.1000514 (PMC2958657; doi:10.1371/journal.pbio.1000514)
Supplement: Table S3 — Functional forms of the components of the cost function for the genetic algorithm. The expression for the total cost is(1)The best possible cost value is 1, when the model results fit all experimental data perfectly. (0.06 MB DOC) [file pbio.1000514.s007.doc]

Supplementary Table S3: Functional forms of the components of the cost function for the genetic algorithm.

The expression for the total cost is $J=\frac{1}{5}\sum_{n=1}^{5} J_{n}+J_{6}$. The best possible cost value is 1 when the model results fit all experimental data perfectly.

| Description | Equation | Variables |
| --- | --- | --- |
| Kinetic flux profiling |  | *N_sp_* = Number of species (55, for both glucose and glutamine labeling)  *N_t_* = Number of experimental time points $M_{i,t}^{calc}$= calculated value for the *i*^th^ metabolite at the *t*^th^ time point $M_{i,t}^{\exp}$= measured laboratory value for the *i*^th^ metabolite at the *t*^th^ time point  *ε_i_* = average of 1 SD of the laboratory measurement for the *i*^th^ metabolite across all time points |
| Uptake and excretion |  | 1: glucose uptake; 2: sum of lactate, alanine, and pyruvate excretion; 3: glutamine uptake; 4: glutamate excretion $F_{i}^{calc}$= the calculated value for the *i*^th^ flux $F_{i}^{\exp}$= the measured laboratory value for the *i*^th^ flux  *ε_i_* = estimated laboratory error for the measurement of the *i*^th^ flux |
| Glycolysis/  PPP ratio |  | $R^{calc}=\frac{3F_{3}}{F_{3}+F_{4}}$ $R^{\exp}=\frac{lactate\_with\_1\_^{13}C\_atom}{lactate\_with\_1\_or\_2\_^{13}C\_atoms}$after 1,2-^13^C-glucose feeding  *ε* = estimated laboratory error |
| Protein Synthesis | $J_{4}=8\times\left\{\begin{matrix} 1:X-X_{\max\leq\varepsilon} \\ \frac{X-X_{\max}}{\varepsilon}:X-X_{\max}>\varepsilon\end{matrix}\right.$ | *X*: calculated protein synthesis rate  *X_max_* = 30 nMoles/min for proliferating cells and 8 nMoles/min for IC7, IC14, and IC14SS7 cells |
| Hexose consumption | $J_{5}=8\times\left\{\begin{matrix} 1:G^{calc}-G^{\max}\leq\varepsilon\\ \frac{G^{calc}-G^{\max}}{\varepsilon}:G^{calc}-G^{\max}>\varepsilon\end{matrix}\right.$ | *G:* calculated hexose phosphate outflux (see Figure S4B)  *G^max^* = 0.2×glucose uptake rate |
| Penalty for negative fluxes | $J_{6}=\sum_{i=1}^{5} \left\{\begin{matrix} 0.5:N_{i}^{calc}<0 \\ 0:N_{i}^{calc}\geq0 \end{matrix}\right\}$ | $N_{i}^{calc}$= calculated net flux value |
